# Canonical Wnt signalling regulates nuclear export of Setdb1 during skeletal muscle terminal differentiation 

**DOI:** 10.1038/celldisc.2016.37

**Published:** 2016-10-18

**Authors:** Sophie Beyer, Julien Pontis, Elija Schirwis, Valentine Battisti, Anja Rudolf, Fabien Le Grand, Slimane Ait-Si-Ali

**Affiliations:** 1 Centre National de la Recherche Scientifique CNRS-Université Paris Diderot, Sorbonne Paris Cité, Epigenetics and Cell Fate UMR7216, Paris, France; 2 Institut Cochin, Université Paris-Descartes, Centre National de la Recherche Scientifique (CNRS) UMR8104, Paris, France; 3 Institut National de la Santé et de la Recherche Médicale (INSERM) U1016, Paris, France

**Keywords:** differentiation, lysine, muscle, methyltransferase, SETDB1/ESET/KMT1E, Wnt signalling

## Abstract

The histone 3 lysine 9 methyltransferase Setdb1 is essential for both stem cell pluripotency and terminal differentiation of different cell types. To shed light on the roles of Setdb1 in these mutually exclusive processes, we used mouse skeletal myoblasts as a model of terminal differentiation. *Ex vivo* studies on isolated single myofibres showed that Setdb1 is required for adult muscle stem cells expansion following activation. *In vitro* studies in skeletal myoblasts confirmed that Setdb1 suppresses terminal differentiation. Genomic binding analyses showed a release of Setdb1 from selected target genes upon myoblast terminal differentiation, concomitant to a nuclear export of Setdb1 to the cytoplasm. Both genomic release and cytoplasmic Setdb1 relocalisation during differentiation were dependent on canonical Wnt signalling. Transcriptomic assays in myoblasts unravelled a significant overlap between Setdb1 and Wnt3a regulated genetic programmes. Together, our findings revealed Wnt-dependent subcellular relocalisation of Setdb1 as a novel mechanism regulating Setdb1 functions and myogenesis.

## Introduction

Tissue regeneration requires precise spatio-temporal regulation of gene expression, which is generally induced by signal transduction pathways. Reconstruction of the adult skeletal muscle tissue relies on a pool of resident committed muscle stem cells located around the myofibres, called the muscle satellite cells (MuSCs). Upon damage to the myofibres, quiescent MuSCs are activated and give rise to a population of transient amplifying myoblasts. Most of the myoblasts then permanently exit the cell cycle, enter terminal differentiation and fuse to form new myofibres to regenerate the tissue, while a subpopulation will re-populate the MuSCs niche [1,2]. Terminal differentiation of skeletal muscle is mainly controlled by a family of specific basic helix–loop–helix transcription factors (including MyoD and Myogenin) that cooperate with members of the MEF2 protein family in the activation of muscle genes [3]. This myogenic transcriptional network acts together with chromatin-modifying regulators, such as histone lysine methyltransferases (KMTs), histone acetyltransferases and histone deacetylases [4]. These enzymes post-translationally modify the amino-terminal tails of histones, which are emerging from the nucleosomes. In particular, histone lysine residues can be tri-, di- or mono-methylated. Depending on the specific histone residue, the degree of methylation and the accessibility mediated by other histone modifications can either positively or negatively regulate gene expression [5]. The KMTs play key roles in transcriptional regulation during development and are also emerging as crucial players in the control of cellular differentiation, including myogenesis [3, 6]. In particular, histone 3 lysine 9 (H3K9) methylation, which is mainly involved in gene repression, is established in euchromatin by G9a/GLP [7] and Setdb1 [8]. Whereas Suv39h1/2 [9] and Prmd3/16 KMTs [10] are essential for the establishment of H3K9 methylation in heterochromatin. H3K9 KMTs are involved in the control of gene expression to regulate cell fate changes [11–13].

Setdb1 (also called Eset and KMT1E) is amplified in melanoma [14] and overexpressed in lung cancer [15]. Setdb1 shows distinct subcellular and subnuclear distributions [16–20] but the meaning of the different Setdb1 localisations remains elusive. Setdb1 is essential in mouse embryonic stem cell (mESC) pluripotency and self-renewal [18, 21, 22] and its knockout is lethal at the peri-implantation stage [23]. Setdb1 is also essential for pluripotency and terminal differentiation of many progenitor cell types. Setdb1 is required for the survival of spermatogonial progenitor cells in mice [24] and a mesenchyme-specific *Setdb1* knockout resulted in an increase of articular chondrocytes terminal differentiation [25]. Furthermore, Setdb1 is crucial for early neurogenesis in mice by promoting proliferation and cell survival [26]. In contrast, Setdb1 regulates osteoblast differentiation during bone development [27] and is involved in the terminal differentiation of growth plate chondrocytes [28]. However, it is unclear how Setdb1 controls these mutually exclusive processes.

To get insights on the apparent opposite functions of Setdb1 in pluripotency versus terminal differentiation, we used skeletal muscle cells as a well-established differentiation model for *in vitro* and *ex vivo* analyses. Our data show that Setdb1 is required for MuSCs expansion following activation and suppresses terminal myoblast differentiation. Furthermore, we demonstrate a nuclear export of Setdb1 during terminal differentiation of myoblasts. Genome-wide studies unravelled that Setdb1 relocalisation is dependent on the canonical Wnt signalling and results in a global release of Setdb1 from its genomic targets and in the de-repression of a subset of Setdb1 target genes. Transcriptomic studies in myoblasts further showed a significant overlap between Setdb1 and Wnt3a regulated genetic programmes. Our results suggest a new regulatory mechanism of Setdb1 by the canonical Wnt signalling pathway to control gene expression in muscle cells.

## Results

### Setdb1 is required for adult skeletal muscle stem cell amplification

**Figure 1:**
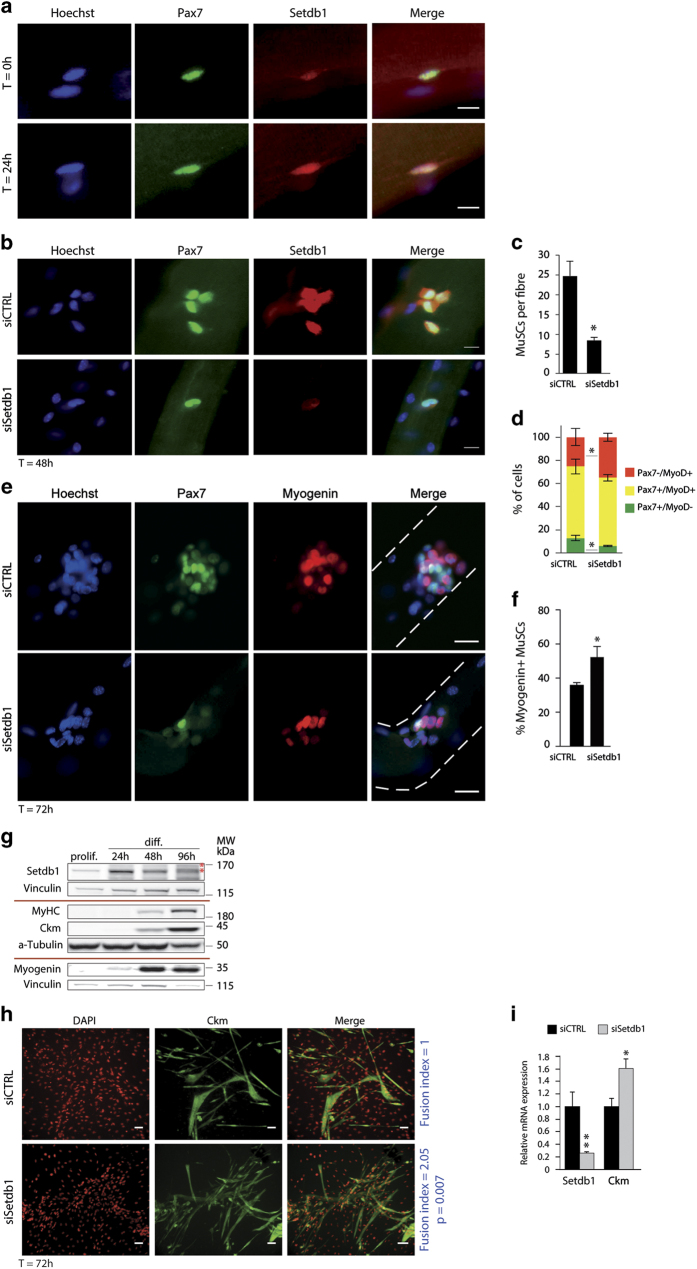
Setdb1 is required for accurate adult skeletal muscle stem cell amplification and negatively regulates terminal differentiation. (**a**) Setdb1 increases during muscle satellite cells (MuSCs) activation. Single myofibres were isolated from extensor digitorum longus (EDL) muscles of C57BL/6N mice. Myofibres were directly fixed after isolation (upper, *T*=0 h) or cultured in floating conditions for 24 h (lower, *T*=24 h). Setdb1 (red) and Pax7 (green) proteins were revealed by indirect immunofluorescence (IF). DNA was labelled with Hoechst (blue). A representative picture of an MuSC is shown. Scale bar=5 μm. (**b**) Setdb1 knockdown in MuSCs. Freshly isolated EDL mouse single myofibres were transfected with control (*siCTRL*) or Setdb1 siRNA (*siSetdb1*) 2 h after isolation and cultured for 48 h post-transfection. IF in MuSCs was done as in (**a**). Scale bar=10 μm. (**c**) Setdb1 knockdown reduces MuSCs amplification. Quantification of MuSCs progeny (Myogenin^+^ or Pax7^+^) on cultured myofibres transfected with control (*siCTRL*) or Setdb1 siRNA (*siSetdb1*) 72 h after transfection. (**d**) Setdb1 knockdown decreases MuSCs self-renewal. Myofibres were transfected as described in (**b**). Quantification (in %) of MuSC descendants at the surface of cultured myofibres 72 h post-transfection. The proportion of committed cells (Pax7^−^/MyoD^+^), proliferating cells (Pax7^+^/MyoD^+^) and self-renewing cells (Pax7^+^/MyoD^−^) in myofibres transfected with control (*siCTRL*) or Setdb1 siRNA (*siSetdb1*) is presented. (**e**) Setdb1 limits MuSCs differentiation. EDL single myofibres were cultured for 72 h following transfection with control (*siCTRL*) or Setdb1 siRNA (*siSetdb1*). For detection of proliferating and differentiating MuSCs indirect IF was performed to detect Pax7 (green) and Myogenin (red), respectively. DNA was labelled with Hoechst (blue). Representative myogenic cell clusters are shown. Scale bar=20 μm. See Supplementary Figure S1A for images with lower magnification. (**f**) Setdb1 knockdown increases the proportion of differentiating MuSCs. % of Myogenin^+^ MuSCs in myofibres transfected with control (*siCTRL*) or Setdb1 siRNA (*siSetdb1*) as described in (**d**). (**g**) Setdb1 protein levels decrease during terminal differentiation of C2C12 myoblasts. Western blot (WB) analysis of Setdb1, Myosin Heavy Chain (MyHC), Creatine Kinase Muscle (Ckm) and Myogenin in whole-cell extracts from proliferating C2C12 myoblasts (prolif.) and after 24, 48 and 96 h of differentiation (diff.). Vinculin served as a loading control for Setdb1 and Myogenin and α-Tubulin for Ckm and MyHC. A typical experiment in shown. * Shifted Setdb1 signals. Images are representative of a minimum of three independent experiments. For Setdb1 signal quantification see Supplementary Figure S1D. (**h**) Setdb1 knockdown promotes differentiation and fusion of myotubes. Proliferating C2C12 myoblasts, at 80–90% confluence, were transfected with control (*siCTRL*) or Setdb1 (*siSetdb1*) siRNA and simultaneously switched to differentiation media for 72 h. Cellular Ckm was revealed by indirect IF (green) and DNA with DAPI (red). Images are representative of a minimum of three independent experiments. Relative fusion index (number of nuclei in myotubes divided by total number of nuclei) is indicated (in blue) for each condition. A minimum of 300 nuclei was counted. Data are presented as mean±s.e.m. of three independent experiments. *P*-values are indicated. Scale bar=20 μm. *See* Supplementary Figure *S1A* *for additional images*. (**i**) Setdb1 knockdown increases *Ckm* mRNA levels. C2C12 cells were transfected with control (*siCTRL*) or Setdb1 siRNA (*siSetdb1*) and differentiated as described in (**h**). Relative mRNA expression of Setdb1 and *Ckm* were represented as fold change relative to *siCTRL* and normalised to *Cyclophilin A* (*CycloA)* and *TATA-box-binding protein* (*TBP*). Data are presented as mean±s.e.m. of three independent experiments. For significance Student paired *t*-test was applied. *P-values <0.05 and are considered significant. **P-values <0.01. For (**a, b, e**): all images are representative of a minimum of three independent experiments using three different mice. For (**c,d**, **f**): data are presented as mean±s.e.m. of three independent experiments using three different mice. For each mouse at least 30 fibres were counted. For significance Student paired *t*-test was applied. *P-values <0.05 and are considered significant. See also Supplementary Figure S1.

We first investigated Setdb1 expression in adult mouse skeletal muscle satellite cells (MuSCs) on single myofibres isolated from the extensor digitorum longus muscles. We used Pax7 (paired box 7 protein) expression to specifically identify MuSCs (Figure 1a–f), as previously described [29]. We detected Setdb1 protein at low levels in quiescent MuSCs in their niche on single fibres immediately after isolation (Figure 1a, top panels). After 24 h in 'floating' culture, Setdb1 was highly expressed in activated dividing MuSCs, mainly in the nucleus but also in the cytoplasm (Figure 1a, bottom panels). Next, we performed Setdb1 loss-of-function assays in MuSCs on isolated myofibres and assayed MuSCs regarding stemness (Pax^+^/MyoD^−^), proliferation (Pax^+^/MyoD^+^) and terminal differentiation (Pax^−^/MyoD^+^ or Pax^−^/Myogenin^+^). Robust and acute Setdb1 knockdown (Figure 1b) reduced MuSCs amplification, as demonstrated by the diminished number of cells per fibre 72 h post-transfection (threefold reduction) (Figure 1c). Extended Setdb1 knockdown led to a higher proportion of cells committing to terminal differentiation (Pax7^−^/MyoD^+^) (Figure 1d, red), and confirmed the reduction in the population that undergoes self-renewal (Pax7^+^/MyoD^−^) (Figure 1d, green). Among the remaining MuSCs we observed a significant increase in Myogenin-expressing cells after Setdb1 knockdown (Figure 1e, f and Supplementary Figure S1A). Together, these results suggested that Setdb1 is positively regulating amplification and negatively affecting terminal differentiation of MuSCs.

We next conducted a series of *in vitro* assays using the C2C12 mouse skeletal myoblast model. We first measured total Setdb1 protein levels in proliferating or differentiating C2C12 myoblasts (Figure 1g). Setdb1 protein was moderately expressed in proliferating myoblasts, peaked early in differentiating myoblasts (24 h of differentiation), decreased again at 48 h and dropped significantly after 96 h of differentiation (Figure 1g and Supplementary Figure S1B). Monitoring the muscle differentiation markers Myogenin, Creatine Kinase Muscle (Ckm) and Myosin Heavy Chain (MyHC) ensured proper cell differentiation (Figure 1g). Besides a significant decrease in Setdb1 total protein after 96 h differentiation, we additionally observed an upward shift in the Setdb1 signal at this time point (Figure 1g, asterisks). This suggests post-translational modifications of Setdb1 at late differentiation. It is possible that cytoplasmic Setdb1 is ubiquitinated and subsequently degraded by the proteasome in late-differentiated myotubes.

**Figure 2:**
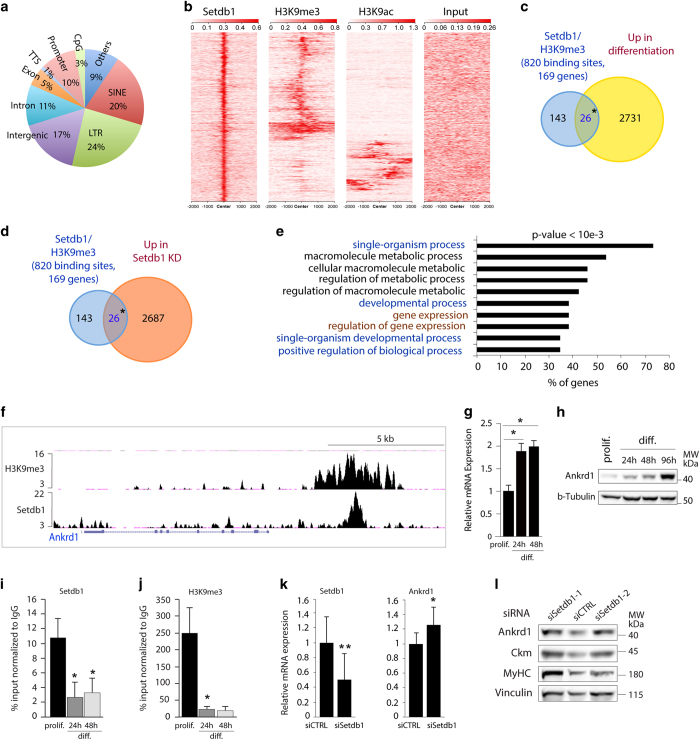
Identification of new Setdb1 target genes, such as *Ankrd1*, in proliferating C2C12 myoblasts. (**a**) Genomic distribution of Setdb1 binding in proliferating C2C12 myoblasts. Enrichments were analysed by ChIP-seq. We performed two independent Setdb1 ChIP-seq experiments (see also Figure 6a) with a Pearson correlation value of 0.7 (not shown). We thus used the most enriched ChIP-seq for peak calling and Genomic. See Supplementary Figure S2A for fold enrichments. (**b**) Heatmap presentation of Setdb1-binding sites (±2 kb from the peak summit) enriched for either H3K9me3 or H3K9ac. Setdb1 and H3K9me3 ChIP-seq were performed in proliferating C2C12 myoblasts. We used MACS software for Setdb1 ChIP-seq analysis and combined MACS and SICER for H3K9me3 ChIP-seq. H3K9ac ChIP-seq was from Asp *et al.* [44] Red intensity corresponds to enrichments, which were subjected to hierarchical clustering. (**c**) Venn Diagram showing the overlap between Setdb1/H3K9me3-enriched genes with upregulated genes in differentiated myotubes. ChIP-seq of Setdb1 and H3K9me3 were performed in proliferating C2C12 myoblasts. Genome-wide data of Setdb1 and H3K9me3-positive genes (±10 kb from TSS) (blue) were crossed with upregulated genes in differentiating myotubes (yellow), from Blais *et al.* [40] For significance Student’s paired *t*-test was applied. *P-values <0.05. (**d**) Venn diagram showing the overlap between Setdb1/H3K9me3-enriched genes with upregulated genes upon Setdb1 knockdown in proliferating myoblasts (*Abca7, Ankrd1, Atxn10, Atxn7l3b, Cd97, Cnn2, Copb1, Fn1, Fstl3, Gnas, Gtf2a1, Hspa2, Irf2, Orai2, Plaur, Prdx1, Prkab2, Ptprs, Rnf215, Srp14, Tmbim6, Tmeff1, Trim24, Tsc22d1, Zfp110, 6430531B16Rik)*. Genome-wide data of Setdb1 and H3K9me3-positive genes (±10 kb from TSS) (blue) were crossed with upregulated genes after Setdb1 acute knockdown (*siSetdb1*) in proliferating C2C12 myoblasts (orange) we measured by RNA-seq. For significance Student’s paired *t*-test was applied. *P-values <0.05*.* (**e**) Gene Ontology (GO) analysis of 26 genes enriched for Setdb1 and H3K9me3 and upregulated after Setdb1 knockdown in proliferating C2C12 myoblasts (from **d**). Preesented are the top 10 biological functions with a *P-*value <10e−3. Fisher's exact test was performed to proof significance. (**f**) Genome Browser presentation of Setdb1- and H3K9me3-binding profiles, analysed by ChIP-seq, at the *Ankrd1* gene in proliferating C2C12 myoblasts. (**g**) *Ankrd1* mRNA levels increase during differentiation in primary myoblasts. Cells were proliferating (prolif.) or differentiated (diff.) for the indicated time (24 or 48 h). Data are represented as fold change relative to proliferation and normalised to *CycloA* and *TBP* mRNA. (**h**) Ankrd1 protein expression increases during C2C12 myoblasts differentiation. Whole-cell extracts from proliferating C2C12 myoblasts (prolif.) or differentiated for the indicated time (24, 48, 72 or 96 h) were analysed by WB. α-Tubulin; loading control. (**i**) Setdb1 and (**j**) H3K9me3 enrichment at the *Ankrd1* enhancer decrease during differentiation in C2C12 myoblasts. ChIP-qPCR results are presented as immunoprecipitated DNA compared with input DNA (% input). Owing to different properties of chromatin compaction during proliferation and differentiation, we normalised specific enrichments (% input) of Setdb1 and H3K9me3 to the corresponding IgGs, which served as a negative control. In addition, according to both our ChIP-Seq data and published ones, we used *COPB1* gene promoter at which Setdb1 binding is constant between proliferating and differentiating; *Nnat* as a positive target and *Ankrd1* regions outside the Setdb1 binding peak as a negative control (not shown). For data presentation as only % input see Supplementary Figure S3D. (**k**) Setdb1 knockdown in differentiating myoblasts increases *Ankrd1* mRNA level. Proliferating C2C12 myoblasts were transfected, at 80–90% confluence, with control siRNA (*siCTRL*) or Setdb1 siRNA (*siSetdb1*) and simultaneously switched to differentiation media for 72 h. Relative mRNA expression levels of Setdb1 and *Ankrd1* were measured. Data are represented as fold change relative to *siCTRL* and normalised to *CycloA* and *TBP* mRNA. (**l**) Setdb1 knockdown decreases Ankrd1 protein level in differentiating C2C12 myoblasts. Cells were treated as described in (**k**). Two specific Setdb1 siRNA were used (*siSetdb1-1*; *siSetdb1-2*) for validation. WB analysis of Setdb1, Ankrd1, Ckm and MyHC were performed in whole-cell extracts. Vinculin served as a loading control. For (**g**, **i**–**k**): Presented data are mean±s.e.m. of a minimum of three independent experiments. For significance Student’s paired *t*-test was applied. *P-values <0.05 and are considered significant. *P*-values <0.01 are marked as **For (**h**, **l**): Images are a representative of a minimum of three different experiments. See also Supplementary Figures S2 and S3.

To investigate the role of Setdb1 during skeletal muscle terminal differentiation *in vitro*, we acutely downregulated Setdb1 using siRNAs at the onset of differentiation in C2C12 myoblasts. We observed increased expression of the late muscle differentiation markers Ckm and MyHC (Figure 1h and i, see Figure 2l for MyHC) and enhanced myotube formation as shown by the significant increase in fusion index in *siSetdb1* condition (Figure 1h, right blue text). To verify that Setdb1 downregulation did not induce aberrant cell death, we performed TdT-mediated dUTP nick-end labelling (TUNEL) stainings in *siCTRL* and *siSetdb1*-transfected cells. We observed that the proportions of cells undergoing programmed cell death were quite low similar between the two conditions and represented only a marginal population (between 6 and 10%) and the difference is not statistically significant (Supplementary Figure S1C). Thus, these data further supported an accelerated differentiation phenotype upon Setdb1 loss-of-function, as in the *ex vivo* myofibre assay, suggesting that Setdb1 impacts skeletal muscle terminal differentiation. After loss-of-function studies we also investigated gain-of-function studies of Setdb1 on the terminal differentiation of myoblasts. To this end, we have established a polyclonal cell population overexpressing Setdb1 by retroviral infection followed by a selection of infected cells using magnetic beads covered by anti-CD25 antibody (as in [30]). C2C12 myoblasts stably overexpressing Setdb1 showed a clear reduction of Myogenin, Ckm and MyHC after 72 h of differentiation compared with the control (Supplementary Figure S1D).

Together, these results indicate that Setdb1 protein levels are crucial in the regulation of the proliferation/differentiation balance in skeletal muscle progenitors.

### Setdb1 pan-genomic distribution in myoblasts and overlap with key histone modifications

To our knowledge, Setdb1 genome-wide localisation is mainly established in mouse ESCs [22, 31, 32], the human lymphoblastic cell line K562 [33] and in a zebrafish melanoma model [14]. To identify Setdb1 target genes in skeletal muscle cells, we performed genome-wide analyses for enrichment of Setdb1 and H3K9me3 by chromatin immunoprecipitation and subsequent DNA sequencing (ChIP-seq) in proliferating C2C12 myoblasts (Supplementary Table S1, and see Figure 2 and Supplementary Figure S2). We tested Setdb1 antibody specificity by conducting ChIP-seq assays in heterozygous versus *Setdb1* KO mESC [34]. ChIP-seq signals were clearly lower in *Setdb1* KO mESC, as exemplified by Setdb1 binding to *Atf7ip2* and *Nnat* genes (Supplementary Figure S2A), strongly suggesting that Setdb1 antibody is specific. We were able to detect 1191 Setdb1-binding sites over the genome (Supplementary Table S1). This number is certainly underestimated since the depth of coverage of our Setdb1 ChIP-seq did not reach any plateau (not shown), and also most likely because of the indirect interaction between this chromatin-modifying enzyme and DNA.

Our ChIP-seq results showed that Setdb1 binds close to genes, especially at core promoter regions and at CpG islands for a significant number of genes (Figure 2a and Supplementary Figure S2B). In addition, a high proportion of Setdb1 binding occurred outside of genes, including approximately 40% DNA repeats (Figure 2a and Supplementary Figure S2B). These observations are in agreement with previous results in mESCs [22, 31, 32].

We next performed different comparisons between our ChIP-seq data sets and the available genome-wide data in C2C12 myoblasts for key histone modifications, such as methylation of lysine 4, 9, 27, 36 and 79 of histone H3 and acetylation of lysine 9, 18 and 27. As expected, we observed a significant H3K9me3, but not H3K27me3, enrichment at Setdb1-binding sites (Figure 2b and Supplementary Figure S2C). Notably, we also detected a large number of Setdb1-binding sites without H3K9me3 enrichment (Figure 2b), as already found by others [14, 22]. Interestingly, a very recent paper by Fei *et al.* [35] described the so-called Setdb1 solo peacks, devoid of H3K9me3 but overlaping with PRC2-binding sites. We cannot exclude that we did not detect H3K9me3 due to technical limitations or the activity of H3K9 demethylases. It is finally possible that H3K9 is only mono or dimethylated due to the absence of the co-factor mAM, which is required for Setdb1 trimethyltransferase activity [36].

Interestingly, we found some common enrichment for the transcriptional activating mark H3K9 acetylation (H3K9ac) and Setdb1, in absence of H3K9me3, suggesting that Setdb1 could bind transcriptionally active regions without maintaining stable H3K9me3 (Figure 2b). Indeed, further analyses confirmed enrichments for active promoter and enhancer histone marks (H3K4me1, H3K4me3, H3 acetylation including acetylated H3K9, H3K18 and H3K27) at Setdb1-binding sites (Supplementary Figure S2C). However, we could not observe strong enrichment for H3K36me3 and H3K79me3 (Supplementary Figure S2C) that mark actively transcribed gene bodies. We thus speculate that Setdb1 could mediate gene transcription activation in some cases, as it was shown for another key H3K9 KMT, G9a [37, 38].

Combined, these pan-genomic data indicate that Setdb1 is mainly associated with transcriptionally silent chromatin, but also to some extent to active regulatory regions, as previously suggested [14].

### Identification of new Setdb1 target genes in myoblasts: the case of *Ankrd1* and *Ataxin 10*

We next asked the functional meaning of the Setdb1/H3K9me3-marked regions in terms of regulating gene expression during muscle terminal differentiation. However, spread histone marks such as H3K9me3 need a particular attention in terms of peak calling. Therefore, in order to uncover the functional meaning of the H3K9me3 ChIP-seq in myoblasts, we used a less stringent peak calling and a SICER peak caller [39], adapted for this type of spread histone marks. Thus, we performed further analyses using the union of SICER and MACS low peak calling. Doing this, we obtained more than 50 000 H3K9me3-enriched regions, which overlap with 820/1 191 peaks (68.8%) of Setdb1-binding sites (Supplementary Figure S2D). The apparent low number of H3K9me3 bound by Setdb1 (820/50 000, 1.3%), also observed by Fei *et al.* [35], could be explained in different manners. First, we have applied a highly stringent bioinformatic analysis to detect Setdb1-binding sites in order to obtain highly relevant binding sites. Second, ChIP-seq assays of histone marks are technically easier to perform compared with chromatin-binding proteins. Therefore, it is not surprising that H3K9me3 ChIP-seq would be more efficient compared with Setdb1. Finally, other H3K9me3 KMTs, such as Suv39h, could also generate, and co-localise with, H3K9me3.

These 820 sites Setdb1/H3K9me3-marked correspond to 169 genes (Figure 2c and d). Among these 169 genes, 26 were upregulated during C2C12 myoblast differentiation (Figure 2c), according to published transcriptomic data [40]. We next performed a transcriptomic assay by RNA-seq after acute Setdb1 knockdown in proliferating myoblasts and established the overlapping profiles between Setdb1/H3K9me3 ChIP-seq and RNA-seq data sets. Among the 169 genes bound by Setdb1 and enriched for H3K9me3, 26 were upregulated upon Setdb1 acute knockdown (Figure 2d). These 26 genes are involved in development, gene expression and metabolic processes (Figure 2e). Interestingly, 7 genes (*Hspa2*, *Prkab2*, *Ankrd1*, *Srp14*, *Fstl3*, *Zfp110 and Ptprs*) are common between the 26 genes in Figure 2c and those in Figure 2d. Two examples of Setdb1/H3K9me3-enriched genes were *Ankrd1* (*Ankyrin repeat domain 1*, Figure 2f) and *Atxn10* (*Ataxin 10*, Supplementary Figure S3A), but neither has previously been defined as a Setdb1 target gene.

Ankrd1 protein (also called CARP for Cardiac Ankyrin Repeat Protein) belongs to the muscle ankyrin repeat protein (MARP) family (including also Ankrd2 and DARP). Ankrd1 appeared of particular interest due to both its structural and regulatory functions in skeletal and cardiac muscles and its involvement in myogenesis [41]. Thus, we further studied its functional relevance in our experimental models.

We first analysed Ankrd1 mRNA and protein levels in proliferating versus differentiating myoblasts. We observed increased *Ankrd1* mRNA levels in primary myoblasts (Figure 2g) and C2C12 myoblasts (Supplementary Figure S3B) at the onset of differentiation. Furthermore, while Ankrd1 protein is already detected in proliferating C2C12 myoblasts, it increases early in differentiating C2C12 myoblasts with a striking increase after 96 h of differentiation (Figure 2h). These findings are consistent with published expression microarray data [40] and with the expression profile of Ankrd1 during muscle tissue regeneration [42].

These observations prompted us to investigate whether the increased Ankrd1 level is due to reduced genomic occupancy of Setdb1 and H3K9me3 during differentiation. Published ChIP-seq data indicate significant enrichment in H3K27ac at the *Ankrd1* proximal promoter and increased RNA pol II recruitment to the transcribed region of *Ankrd1* (Supplementary Figure S3C) [43–45]. Thus, we performed ChIP-qPCR at the *Ankrd1* enhancer to measure Setdb1 binding and H3K9me3 during terminal differentiation. We confirmed Setdb1 and H3K9me3 enrichment at the *Ankrd1* enhancer in proliferating C2C12 myoblasts and we observed a significant reduction at the onset of differentiation (Figure 2i and j and Supplementary Figure S3D). Furthermore, we observed a tendency to increased H3K9ac at the *Ankrd1* enhancer after 24 h of differentiation (Supplementary Figure S3E). It is thus possible that the removal of Setdb1/H3K9me3 is required to prime the *Ankrd1* enhancer for recruiting other factors and subsequently RNA pol II to the promoter (Supplementary Figure S3C). Longer differentiation times did not cause a further decrease in Setdb1 and H3K9me3 occupancy (Figure 2i and j and Supplementary Figure S3D, 48 h), suggesting that the release from the enhancer of *Ankrd1* is an early event preceding its expression in differentiating myoblasts (as shown in Figure 2g and h and Supplementary Figure S3B). To confirm that *Ankrd1* is a functional target of Setdb1 in C2C12 myoblasts, we downregulated Setdb1 at the onset of differentiation (as in Figure 1h) and observed an increase in *Ankrd1* mRNA (Figure 2k) and protein (Figure 2l) levels compared with control cells. Similarly, *Atxn10* expression is also increased during myoblast differentiation [40] and we observed decreased occupancies of Setdb1 and H3K9me3 at the *Atxn10* promoter (Supplementary Figure S3F). Interestingly, Ankrd1 and Atxn10 mRNA increased also upon Setdb1 knockdown in proliferating C2C12 myoblasts (Supplementary Table S2), further confirming their regulation by Setdb1.

Together, these data suggest that, at the onset of terminal differentiation, the removal of Setdb1/H3K9me3 at the *Ankrd1* enhancer could be pivotal in *Ankrd1* activation.

### Ankrd1 is crucial for myoblast terminal differentiation

**Figure 3:**
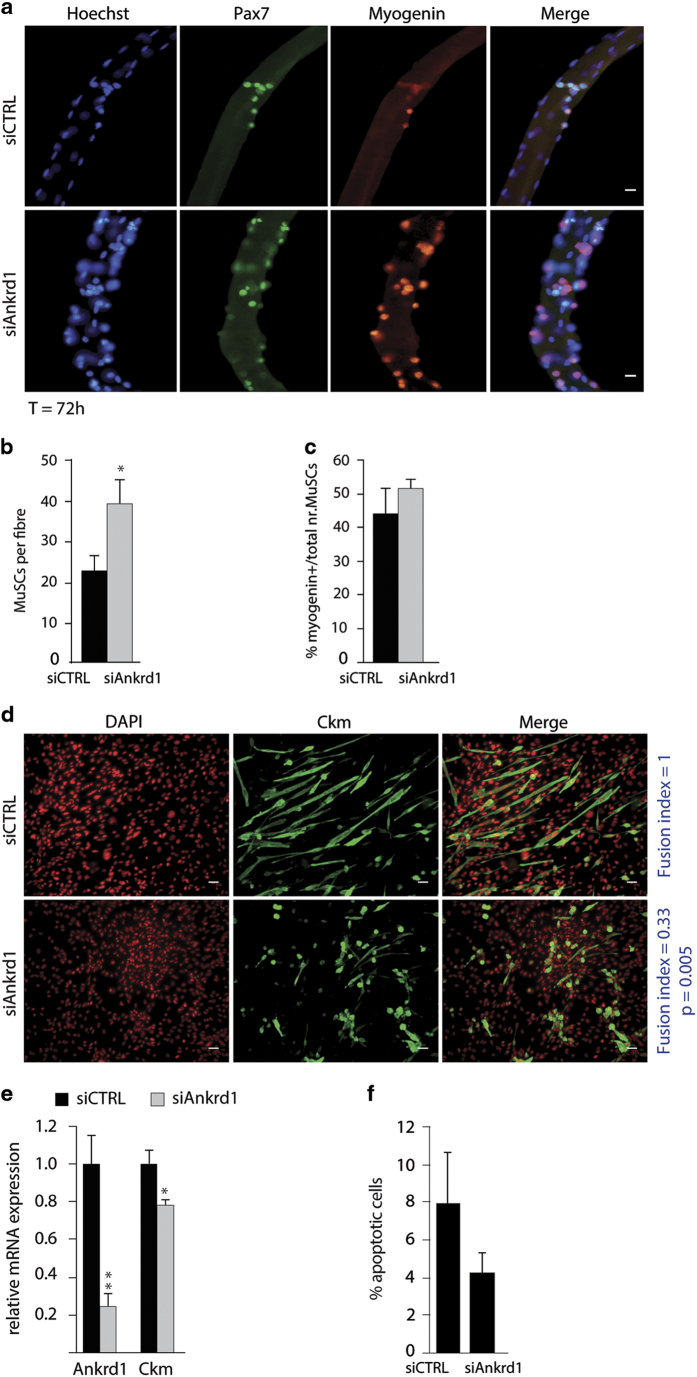
The newly identified Setdb1 target *Ankrd1* is crucial for myoblast terminal differentiation. (**a**) Downregulation of Ankrd1 increases MuSC amplification in single myofibres. Control siRNA (*siCTRL*) or Ankrd1 siRNA (*siAnkrd1)* were transfected directly after isolation and cultured in suspension for 72 h. Indirect IF was conducted to visualise Pax7 (green) and Myogenin (red) in MuSCs. DNA was detected with Hoechst (blue). Scale bar=5 μm. (**b**) Quantification of MuSCs progeny (Pax7^+^ or Myogenin^+^) in myofibres transfected with control siRNA (*siCTRL*) or Ankrd1 siRNA (*siAnkrd1*). (**c**) Quantification of differentiating MuSCs (Myogenin+) in myofibres transfected with control siRNA (*siCTRL*) or Ankrd1 siRNA (*siAnkrd1*). (**d**) Ankrd1 knockdown impairs myotube formation. Proliferating C2C12 myoblasts, at 80–90% confluence, were transfected with control siRNA (*siCTRL*) or Ankrd1 siRNA (si*Ankrd1*) and simultaneously switched to differentiation media for 72 h post-transfection. Cellular Ckm was stained by indirect IF (green). DNA was co-stained with DAPI (red). Scale bar=20 μm. Fusion index (number of nuclei in myotubes divided by total number of nuclei) is indicated (in blue) for each condition. A minimum of 300 nuclei was counted. Data are presented as mean±s.e.m. of *three* independent experiments. *P*-values are indicated. (**e**) Ankrd1 downregulation reduces *Ckm* mRNA levels. Proliferating C2C12 myoblasts were transfected and cultured as described in (**d**). Relative mRNA expression levels of *Ankrd1* and *Ckm* were evaluated by qPCR. Data are represented as fold change relative to *siCTRL* and normalised to *CycloA* and *TBP*. (**f**) Proliferating C2C12 myoblasts were transfected and differentiated as described in (**d**). Apoptotic cells were stained by performing the TdT-mediated dUTP-biotin nick-end labelling (TUNEL) reaction. Cells containing a signal inside the nucleus were considered as apoptotic. A minimum of 400 cells was counted. For *(***a**, **d**): Images are representative of at least three independent experiments. For (**b, c**): Presented data are mean±s.e.m. of four independent experiments using four different mice. For each experiment at least 25 fibres were evaluated. For significance Student’s paired *t-*test was applied. *P-values <0.05 are considered significant. For (**e**, **f**)*:* Presented data are mean±s.e.m. of a minimum of three independent experiments. For significance Student’s paired *t*-test was applied. *P-values <0.05 are considered significant.

We next tested the functional role of Ankrd1 in skeletal muscle cell fate. First, we investigated its relevance in MuSC proliferation on single myofibres (Figure 3a). According to Pax7 and Myogenin stainings, Ankrd1 knockdown on single myofibres generated larger clusters of amplifying MuSCs progeny at the surface of their host myofibres compared with control MuSCs (Figure 3a and b). Importantly, this was not related to a block in MuSC differentiation since quantification of the numbers of Myogenin+ cells showed that the proportion of differentiating cells remained similar in both conditions (Figure 3c). In conclusion, reduced Ankrd1 expression seems to promote MuSC amplification *ex vivo*.

We next investigated Ankrd1 functions during C2C12 myoblasts terminal differentiation. We downregulated Ankrd1 at the onset of differentiation and observed a reduction in Ckm level (Figure 3d and e). Furthermore, *siAnkrd1*-transfected myoblasts showed a strong and significant impairment in the ability to fuse and form myotubes (Figure 3d and Supplementary Figure S3G), with a fusion index of 0.33 compared with control (Figure 3d, right, in blue). We also performed TUNEL assays and observed that *siAnkrd1* did not significantly induce apoptosis compared with control-transfected cells (Figure 3f). Thus, we highlighted the role of the newly identified Setdb1 target Ankrd1 in promoting skeletal muscle cell differentiation.

### Setdb1 subcellular localisation changes upon terminal muscle differentiation in an Exportin-1-dependent manner

**Figure 4:**
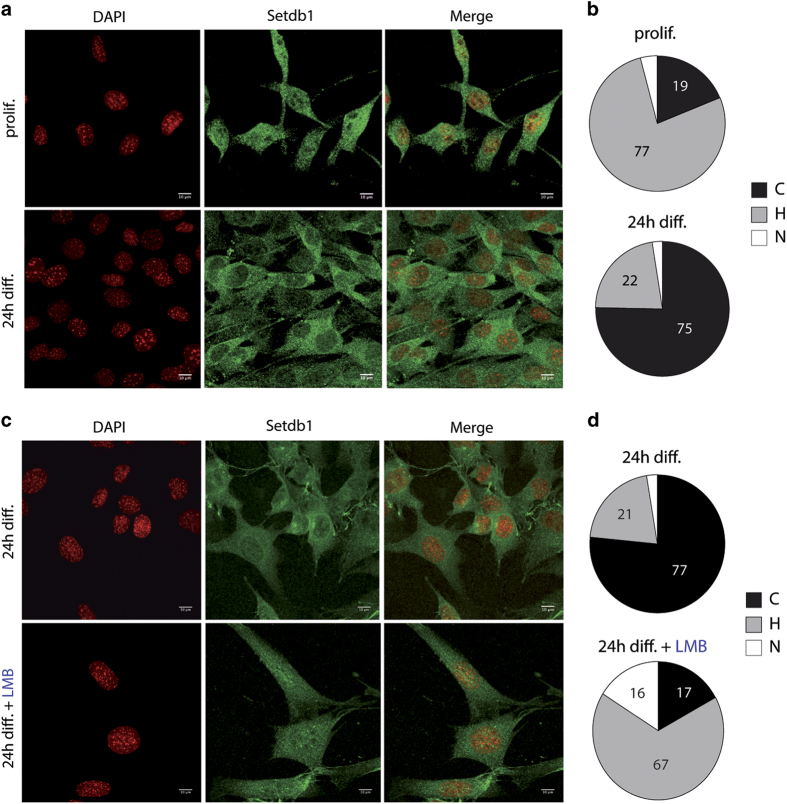
Setdb1 subcellular localisation changes upon terminal muscle differentiation and is dependent on Exportin-1. (**a**) Setdb1 localisation changes in C2C12 myoblasts during differentiation. Cells were proliferating (prolif.) or differentiating for 24 h (24 h diff.). Cellular Setdb1 (green) was detected by indirect IF. Cells were DAPI-stained to reveal DNA (red) prior to confocal fluorescent microscopy analyses. Scale bar=10 μm. (**b**) Quantification of Setdb1 localisation in proliferating (prolif.) and differentiating (24 h diff.) C2C12 myoblasts. Setdb1 localisation was classified as nuclear *(*N), homogeneous (H), or cytoplasmic (C)*.* A minimum of 100 cells was counted for each condition. (**c**) Setdb1 delocalisation in differentiating C2C12 myoblasts is restricted by blocking nuclear export. Indirect IF of Setdb1 (green) and confocal microscopy were performed as described in (**a**). C2C12 myoblasts were differentiated for 24 h and non-treated (24 h diff.) or incubated in parallel with LMB for the last 18 h (24 h diff.+LMB). Scale bar=10 μm. (**d**) Quantification of Setdb1 localisation in C2C12 myoblasts differentiated for 24 h and non-treated (24 h diff.) or incubated in parallel with LMB for the last 18 h (24 h diff.+LMB). Setdb1 localisation was classified as described for (**b**). A minimum of 100 cells was counted for each condition. For (**a**, **c**): Images are representative of a minimum of three independent experiments. For (**b**, **d)**: Data are presented as mean±s.e.m. of a minimum of three independent experiments. See also Supplementary Figure S4.

Since de-repression of Setdb1 target genes is instrumental in skeletal muscle cell transition from proliferation to differentiation and since this process is not due to a decrease in Setdb1 protein levels in early differentiation (Figure 1g and see below Supplementary Figure S5C and D), we hypothesised that a redistribution of Setdb1 subcellular localisation could explain these observations. Setdb1 was reported to be both nuclear and cytoplasmic in human cells and in *C. elegans* [19, 20, 46, 47]. The function and regulation of this subcellular localisation remain largely unknown. We thus sought to investigate whether Setdb1 subcellular localisation changes during skeletal muscle terminal differentiation and if this phenomenon could regulate its functions. We first confirmed Setdb1 nuclear and cytoplasmic localisation in C2C12 myoblasts (Supplementary Figure S4A and B and see Materials and Methods for more details) and in several other human and mouse cell lines (HeLa, ESCs, MEFs and MRC5; Supplementary Figure S4C). We have checked the specificity of the Setdb1 cytoplasmic Immunofluorescence (IF) signal by both using antibodies from different companies (not shown), and gain or loss-of-function assays showing that the Sedtb1 cytoplasmic signal is proportional to the Setdb1 levels (Supplementary Figure S4A). Our data showed that in proliferating C2C12 myoblasts Setdb1 is homogeneously (H) distributed between the cytoplasm and the nucleus in 77% of cells (Figure 4a and b). However, 19% of proliferating myoblasts already exhibited a dominant cytoplasmic localisation (C), with only a minor proportion (4%) showing predominantly nuclear Setdb1 (N) (Figure 4b). In contrast, in differentiating C2C12 myoblasts up to 75% of cells showed a mainly cytoplasmic Setdb1 (Figure 4a and b) and only a small portion had either a homogeneous (22%) or dominant nuclear (2%) localisation (Figure 4b).

We next investigated whether the cytoplasmic localisation of Setdb1 was due to nuclear export. Exogenous Setdb1 was reported to be mainly cytoplasmic and shifted to the nucleus when export was blocked by Leptomycin B (LMB) [17], which inhibits nuclear export via the principal nuclear export pathway involving Exportin-1 [48]. Here, LMB treatment decreased cytoplasmic localisation of Setdb1 during myoblast differentiation from 77% to 17% of cells (Figure 4c and d). Furthermore, proliferating C2C12 myoblasts (Supplementary Figure S4D) and HeLa cells (Supplementary Figure S4E), incubated with LMB, showed a higher nuclear localisation of Setdb1 compared with the homogeneous distribution in untreated cells. Global Setdb1 protein levels were unaffected (Supplementary Figure S4F) and the efficiency of LMB treatment was controlled by visualising the block of nuclear export of the NFκB subunit RelA [49] (Supplementary Figure S4G).

We additionally performed subcellular fractionation of proliferating and differentiating C2C12 myoblasts. We detected Setdb1 in the nuclear and the cytoplasmic fraction in proliferating cells as well as after 24 h differentiation (Supplementary Figure S4H). We, however, observed a tendency of enrichment in the ratio of cytoplasmic versus nuclear Setdb1 in differentiating myoblasts. We noticed the appearance of new (iso)-forms of Setdb1 in the cytoplasm, probably due to post-translational modifications. These new bands might correspond to Setdb1 since they disappear after Setdb1 siRNA-mediated knockdown (not shown). Further studies must be performed to identify these Setdb1 post-translational modifications.

In summary, we propose that Setdb1 is actively exported from the nucleus via an Exportin-1-dependent mechanism. Besides a significant decrease in Setdb1 total protein after 96 h differentiation, we additionally observed an upward shift in the Setdb1 signal at this time point (Figure 1g, asterisks). This suggests post-translational modifications of Setdb1 at late differentiation. It is possible that cytoplasmic Setdb1 is ubiquitinated and subsequently degraded by the proteasome in late-differentiated myotubes.

### Setdb1 cellular relocalisation is dependent on Wnt3a signalling

**Figure 5:**
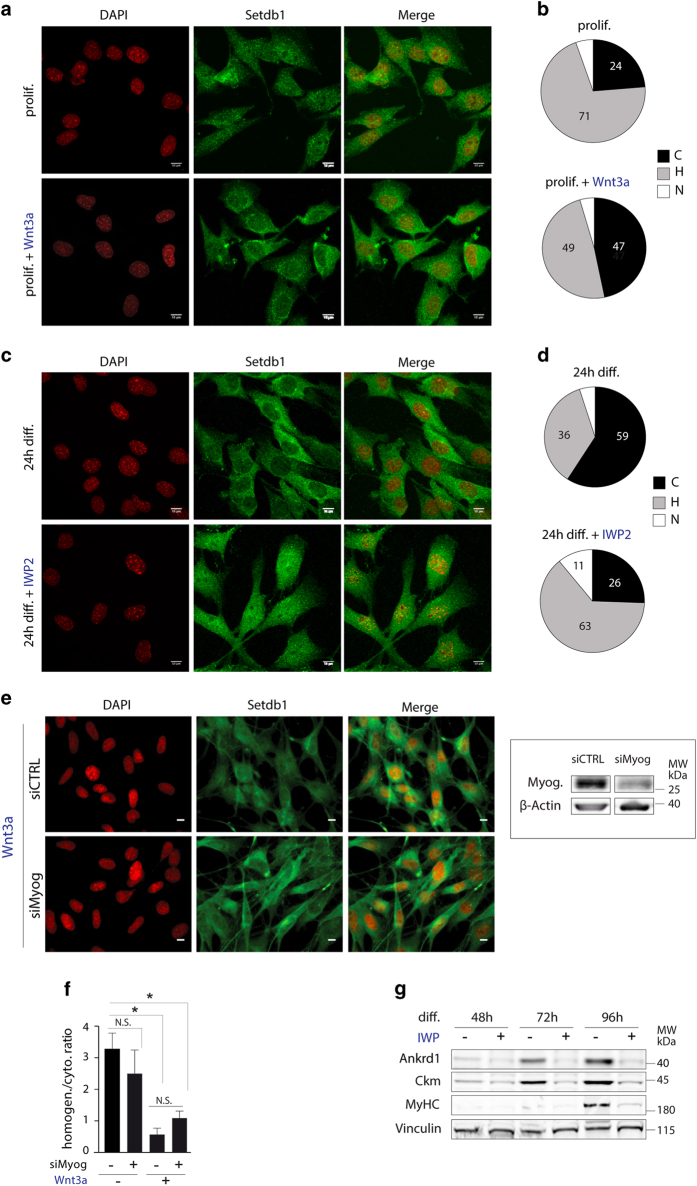
Setdb1 cellular relocalisation upon terminal differentiation is dependent on Wnt3a signalling. (**a**) Setdb1 localisation changes in C2C12 myoblasts with increased Wnt3a signalling. Indirect IF and confocal microscopy of Setdb1 (green) was conducted in proliferating C2C12 myoblasts (prolif.) and stimulated with Wnt3a protein for 24 h (prolif.+Wnt3a). DNA was stained with DAPI (red). Scale bar=10 μm. (**b**) Quantification of a minimum of 100 cells described in (**a**) according to their phenotype. Setdb1 localisation is cytoplasmic (C), homogeneous (H) or mainly nuclear (N). (**c**) Setdb1 delocalisation is restricted in differentiating C2C12 myoblasts when Wnt signalling is inhibited. Indirect IF of Setdb1 as explained in (**a**). C2C12 myoblasts were differentiating for 24 h (24 h diff.) and treated with IWP2 (24 h diff.+IWP2), an inhibitor of Wnt production, in parallel for the same period of time. (**d**) Quantification of a minimum of 100 cells described in (**c**) according to their phenotype. Setdb1 localisation is cytoplasmic (C), homogeneous (H) or mainly nuclear (N). (**e**) Wnt3a signalling is sufficient for Setdb1 delocalisation in C2C12 myoblasts. Left panels: Proliferating cells, at 80–90% confluence, were transfected with control siRNA (*siCTRL*) or Myogenin siRNA (*siMyog*) and simultaneously switched to differentiation media for 24 h. Additionally, cells were treated with Wnt3a. Indirect IF was performed, as described in (**a**). Scale bar=2 μm. Right panel: knockdown efficiency of Myogenin was analysed in parallel in whole-cell extracts by WB. α-Tubulin, loading control. (**f**) Wnt3a signalling is sufficient for Setdb1 delocalisation in primary myoblasts. Proliferating cells were transfected with control (*siCTRL*) or Myogenin siRNA (*siMyog*) and concomitantly stimulated by Wnt3a protein (+Wnt3a) for 24 h. Setdb1 localisation was classified as homogeneous (homogen.) or cytoplasmic (cyto.) and the ratio was calculated. A minimum of 100 cells was counted for each condition. (**g**) Inhibition of Wnt signalling reduces Ankrd1, Ckm and MyHC levels. WB was performed in whole-cell extracts from C2C12 cells, differentiated (diff.) for the indicated time (in **h**) and simultaneously treated with IWP2. Vinculin; loading control. For (**a**, **c, e** and **g**): Images are representative of a minimum of three independent experiments. For (**b** and **d**): Data are presented as mean of a minimum of three independent experiments. For (**f**): Data are presented as mean±s.e.m. of a minimum of three independent experiments. For significance Student’s paired *t*-test was applied. *P-values <0.05 are considered significant. See also Supplementary Figure S5.

Canonical Wnt signalling is critical for embryonic myogenesis [50, 51] and has been functionally linked to MuSCs and C2C12 differentiation [50, 52, 53]. Wnt/β-Catenin also regulates *Ankrd1* expression in mouse tumours [54]. We thus decided to test Wnt signalling involvement in Setdb1 relocalisation during myoblast differentiation. To this aim, we used recombinant Wnt3a proteins to artificially increase canonical Wnt signalling in proliferating myoblasts, and thus mimic the endogenous increase in Wnt3a level documented in differentiating myoblasts [55]. Activation of canonical Wnt signalling by recombinant Wnt3a induced a preferential redistribution of Setdb1 to the cytoplasm in proliferating myoblasts (Figure 5a), reminiscent of cytoplasmic relocalisation of Setdb1 in differentiating myoblasts (Figure 4). Whereas only 24% of untreated proliferating myoblasts showed a cytoplasmic Setdb1 localisation, 47% of proliferating myoblasts had a Wnt3a-induced dominant cytoplasmic localisation of Setdb1 (Figure 5b).

To exclude that Setdb1 relocalisation is due to terminal differentiation and that the Wnt3a-dependent relocalisation is a secondary effect, we tested whether inhibiting endogenous Wnt signalling would abrogate Setdb1 relocalisation to the cytoplasm during terminal differentiation. We blocked Wnt signalling in differentiating myoblasts using IWP2, a small molecule inhibitor of Wnt production [56]. As expected, 59% of untreated differentiating myoblasts had Setdb1 mainly in the cytoplasm, whereas only 26% of IWP2-treated myoblasts showed this phenotype (Figure 5c and d). Instead, 63% of IWP2-treated differentiating myoblasts displayed homogeneous Setdb1 localisation and 11% had Setdb1 mainly in the nucleus (Figure 5c and d). We observed concordant effects of Wnt3a and IWP2 treatments in proliferating and differentiating MuSC-derived primary myoblasts, respectively (Supplementary Figure S5A and B).

To ensure that our observations were not due to Setdb1 degradation, we analysed Setdb1 protein levels. Besides the previously observed increase during early differentiation (Figure 1g), neither Wnt3a nor IWP2 treatments showed effects on total Setdb1 protein level (Supplementary Figure S5C for C2C12 myoblasts and Supplementary Figure S5D for MuSCs). Analyses of active β-Catenin protein levels demonstrated activation of canonical Wnt signalling both upon exogenous Wnt3a ligand treatment and in differentiating cells (Supplementary Figure S5C and D). Finally, we observed a decrease in the early differentiation marker Myogenin after IWP2 treatment in C2C12 myoblasts, confirming perturbation of terminal differentiation when Wnt signalling is blocked (Supplementary Figure S5C).

Efficacy of Wnt3a and IWP2 treatments was further confirmed by quantification of the Wnt3a *bona fide* target gene *Axin2* [57], which was activated by Wnt3a and inhibited by IWP2 (Supplementary Figure S5E). Analysis of the proliferation marker *Ccnd1* (encoding Cyclin D1) showed that cells exit the cell cycle independently of the treatments (Supplementary Figure S5E).

To further demonstrate that Wnt3a mediates Setdb1 relocalisation, we prevented C2C12 myoblasts (Figure 5e) or primary myoblasts (Figure 5f) to differentiate by reducing Myogenin at the onset of differentiation but cultured them in differentiation conditions. In parallel, cells were stimulated with Wnt3a throughout the entire experimental setup. Our results showed that the removal of Setdb1 from the nucleus following Wnt3a stimulation was comparable regardless of Myogenin levels (and differentiation) indicating that canonical Wnt signalling alone is sufficient to relocate Setdb1 to the cytoplasm (Figure 5e and f). Efficient downregulation of Myogenin by siRNA transfection was analysed at the protein level (Figure 5e, right panel). Elevated *Axin2* mRNA expression levels and increased β-Catenin nuclear accumulation after addition of Wnt3a confirmed activation of canonical Wnt pathway in the absence of Myogenin expression (data not shown).

To further show that Setdb1 relocalisation is dependent on Wnt3a, we performed additional experiments in HeLa cells. Likewise, we detected a relocalisation of Setdb1 to the cytoplasm upon Wnt3a treatment and an increased nuclear localisation when Wnt secretion was blocked by IWP2 (Supplementary Figure S5F and G). These data suggested that Setdb1 export to the cytoplasm is dependent on canonical Wnt signalling even in cells that do not differentiate. Additionally, it indicates that this is a conserved mechanism among cells of different tissues and species.

Furthermore, we observed reduced expression of the Setdb1 target Ankrd1, along with differentiation markers Ckm and MyHC, when C2C12 cells were cultured in differentiation conditions up to 96 h and treated with IWP2 (Figure 5g). These results show that an active Wnt3a signalling during differentiation is required for the expression of the Setdb1 target Ankrd1. We also demonstrate that proper activation of canonical Wnt signalling is required for the appropriate differentiation of C2C12 myoblasts.

Since Setdb1 is relocated to the cytoplasm during differentiation, we investigated if β-Catenin is simultaneously relocated to the nucleus. We observed an increased β-Catenin signal in the nucleus in primary myoblasts (Supplementary Figure S5H) and C2C12 myoblasts (data not shown) after 24 h of differentiation compared with proliferating cells. This observation is in line with previously reported work regarding β-Catenin and its translocation to the nucleus during differentiation or due to increased canonical Wnt signalling [58].

Collectively, our data show that the cytoplasmic localisation of Setdb1 is dynamically regulated by the canonical Wnt pathway in cells of different origins. They further demonstrate the relevance of active Wnt signalling for the expression of Setdb1 targets in myoblasts and myogenic differentiation.

### Wnt3a induced change of Setdb1 occupancy at certain target gene promoters

**Figure 6:**
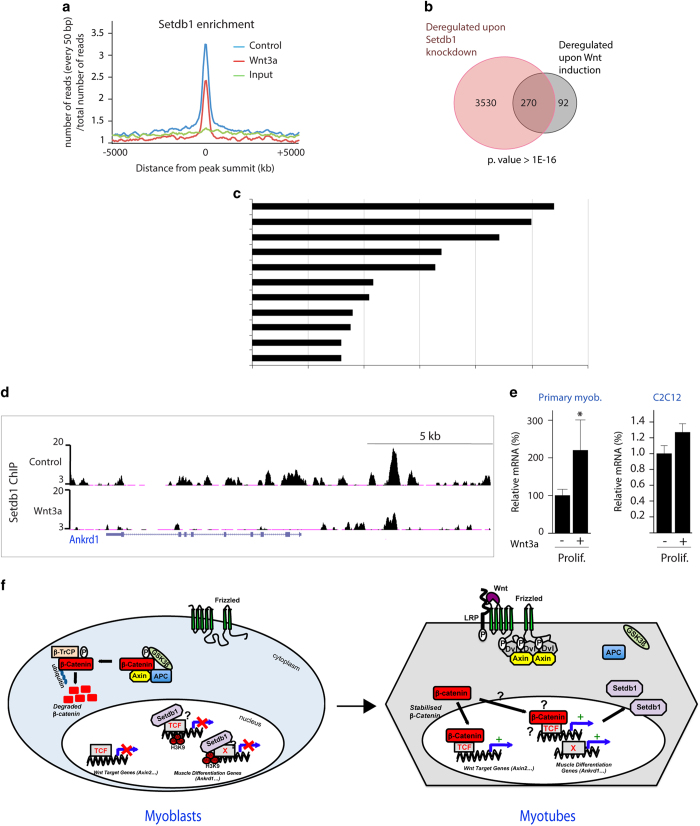
Wnt3a changes occupancy of Setdb1 at certain target gene promoters. (**a**) Setdb1 genome-wide recruitment is reduced after Wnt3a stimulation. ChIP-seq was performed in proliferating C2C12 myoblasts non-treated (Control) or treated with Wnt3a (Wnt3a). Graphic presents average Setdb1 binding density of genomic regions surrounding (±5 kb) Setdb1-binding sites (as in Figure 2a, FDR<1%, fourfold enrichment over the input and *P*-value<10^−5^). Setdb1 occupancy and input density are plotted as average of reads density (every 50 bp) and normalised to total number of reads. (**b**) Setdb1 and canonical Wnt signalling have common target genes in myoblasts. Deregulated genes upon Setdb1 acute knockdown in proliferating C2C12 myoblasts (red), analysed by RNA-sequencing, were crossed with deregulated genes upon Wnt3a stimulation in proliferating primary myoblasts (grey), analysed by microarray. Results are presented as Venn diagram. The correlation is determined as highly significant by hypergeometric test. *P*-value<10^−16^. (**c**) GO analysis of 245 (out of 270) commonly (in the same way) deregulated genes after Setdb1 acute knockdown in proliferating C2C12 myoblasts and Wnt3a stimulation in primary myoblasts as described in (**b**). Fisher's exact test was performed to proof significance. *P*-value between 10^−30^ and 10^−100^. (**d**) Genome Browser presentation of Setdb1-binding profile at the *Ankrd1* enhancer in proliferating C2C12 myoblasts non-treated (control) or treated with Wnt3a for 24 h (Wnt3a). (**e**) Wnt3a increases Ankrd1 expression in proliferating primary and C2C12 myoblasts. Cells were untreated or stimulated with Wnt3a protein for 24 h (primary myoblasts) and 50–72 h (C2C12 myoblasts). Relative mRNA expression analysis of *Ankrd1* was performed. Data are represented as fold change relative to untreated cells and normalised to *CycloA* and *TBP*. Data are presented as mean±s.e.m. of three independent experiments. For significance Student paired *t*-test was applied. *P-values <0.05 and are considered significant. (**f**) Model of Wnt3a-dependent nuclear export of Setdb1 and subsequent gene activation. In myoblasts (left) Setdb1 is located in the nucleus, where it occupies its target genes, such as *Ankrd1*, and represses their transcription by methylating H3K9. Hereby, interaction with other proteins are likely and require further investigation. When canonical Wnt signalling is repressed, β-Catenin is phosphorylated and degraded. In myotubes (right) Setdb1 is released from certain target genes and exported from the nucleus in a Wnt3a-dependent manner. Owing to the lack of H3K9me3 a subset of target genes are transcribed. It is possible that Setdb1 is replaced at certain promoters/enhancers by β-Catenin due to its translocation to the nucleus. The exported Setdb1 could be marked for nuclear export by certain post-translational modifications, such as phosphorylation. Proteasomal degradation of Setdb1 in the cytoplasm is possible. P, phosphrylation; X, any transcription factor; me in red circle, methylation. See also Supplementary Figure S6.

To test whether Wnt3a-mediated cytoplasmic relocalisation of Setdb1 impacts its genomic targeting, we conducted Setdb1 ChIP-seq in proliferating C2C12 myoblasts treated or not with Wnt3a. We observed a global decrease in Setdb1 pan-genomic binding in myoblasts upon Wnt3a treatment (Figure 6a), concomitant to the Wnt3a-induced nuclear export of Setdb1 (described in Figure 5). Interestingly, when we crossed our RNA-seq data of Setdb1 knockdown (Supplementary Table S2) with microarray data of proliferating myoblasts treated with Wnt3a [59], we obtained a highly significant overlap of commonly deregulated genes (Figure 6b). Indeed, 270/362 (74.5%) among Wnt3a-responsive genes were also deregulated upon Setdb1 knockdown (Figure 6b). These co-regulated genes are mainly involved in cell cycle, mitosis and cellular component organisation (Figure 6c). These findings further consolidate the functional link between Wnt3a signalling and Setdb1.

As expected, we found again *Ankrd1* (Figure 6d) and *Atxn10* (Supplementary Figure S6A) among these loci with decreased Setdb1 occupancy upon Wnt3a treatment. Accordingly, we observed increased *Ankrd1* expression in proliferating MuSC-derived primary and in C2C12 myoblasts in response to Wnt3a (microarray analysis [60], and Figure 6e). Likewise, exogenous Wnt3a strengthened the *Ankrd1* upregulation also in differentiating myoblasts (Supplementary Figure S6B and C). Our results are in agreement with a previous work demonstrating that *Ankrd1* is upregulated in mammary tumours in a Wnt/β-Catenin-dependent manner [54].

To further investigate the link between Setdb1 and Ankrd1 functions, we analysed the expression of Ankrd1-related genes in Setdb1 knockdown conditions. We thus checked the expression of the Ankrd1 partner Titin [41] and the Ankrd1 target Mmp13 [61]. Interestingly, our RNA-seq data showed that the *Titin* mRNA is strongly decreased while the *Mmp13* mRNA is increased upon Setdb1 knockdown (Supplementary Figure S6D). This further highlighted a functional link between Setdb1 and the 'Ankrd1 pathway'.

Taken together, these data demonstrated that canonical Wnt signalling affects genomic targeting of Setdb1 at a subset of genes. More specifically, decreased Setdb1 occupancy at the *Ankrd1* enhancer is accompanied by increased expression level and myogenic differentiation.

## Discussion

Canonical Wnt signalling has previously been implicated in MuSC physiology and muscle tissue repair. In fact, ectopic activation of the pathway by direct injection of recombinant Wnt3a proteins [55] or by electroporation of a Wnt3a-expressing plasmid [62] both resulted in impaired myofibre repair. Further, misregulation of canonical Wnt signalling was shown to be instrumental in the loss of stem cell potential in MuSCs during ageing [63] and in the pathophysiology of oculopharyngeal muscular dystrophy [64]. Yet, the molecular mechanisms downstream of canonical Wnt signalling in MuSCs and the physiological roles of this pathway during adult myogenesis remained largely unknown.

Here, we identified a novel functional link between canonical Wnt signalling and the major H3K9 KMT Setdb1. We, in addition, established a functional role for Setdb1 in allowing muscle progenitor amplification and preventing skeletal muscle terminal differentiation. We thus propose that canonical Wnt signalling controls MuSCs function, and their amplification following activation, by regulating Setdb1 subcellular localisation and genome occupancy. We showed that the nuclear export of Setdb1 and its genomic release from certain target loci during muscle terminal differentiation are actively regulated by canonical Wnt signalling (Figure 6f). This is crucial for myoblasts to activate the differentiation programme and ultimately form multinucleated myotubes.

Furthermore, it is known that the β-Catenin transcription complex facilitates H3K4 tri-methylation, both globally and at Wnt target loci, via direct binding to H3K4me3 and recruiting KMT complexes [65]. Yet, it has never been previously shown that canonical Wnt signalling activation could functionally interact with an H3K9 KMT. Our findings indicate the need for future global elucidation of epigenetic changes in response to extrinsic signals, such as Wnt(s), in muscle progenitors and other types of stem cells.

While revising our manuscript, a recent paper by Song *et al.* [66] claimed that Setdb1 knockdown in C2C12 proliferating myoblasts delayed terminal differentiation, whereas ectopic Setdb1 expression had only little effect. Thus, in order to study the role of Setdb1 in differentiation entry, we performed Setdb1 knockdown (KD) in confluent myoblasts in differentiation conditions.

In this study, Song *et al.* performed Setdb1 stable KD in proliferating C2C12 myoblasts and this impaired their terminal differentiation. Indeed, C2C12 myoblasts must first exit cell cycle then enter terminal differentiation. Some H3K9 KMTs are required for the cell cycle exit to silence proliferation genes [11–13]. Thus, KD of one of them in proliferating C2C12 could impair cell cycle exit and, subsequently, differentiation entry. Thus, in our study we specifically aimed to discriminate effects on cell cycle exit versus differentiation entry, that is why we performed acute KD of Setdb1 at the onset of terminal differentiation, but not in proliferation conditions. Our results show that Setdb1 loss of function at the proliferation-to-differentiation switch accelerates differentiation. However, our Setdb1 gain-of-function in C2C12 myoblasts severely impaired terminal differentiation compared with the results of Song *et al.* probably due to the different extents of ectopic Setdb1 expression levels.

It is thus possible that Setdb1 is involved both in the cell cycle exit and the fine-tuning of the differentiation kinetics (by regulating, for example, *Ankrd1*, a mid-to-late differentiation marker). In summary, Song *et al.* and our work address two different aspects of Setdb1 in skeletal muscle cell fate regulation. While Song *et al.* showed the importance of Setdb1 in proliferating myoblasts, we focused our work on the relevance of Setdb1 at the onset of muscle terminal differentiation, right after cell cycle exit.

Several studies have identified Setdb1 in the cytoplasm [19] and its cytoplasmic methyltransferase activity was demonstrated both in mammals [46] and *C. elegans* [47]. Furthermore, Setdb1 binds several non-histone proteins in the nucleus [22, 67] and can also methylate some of them [68, 69]. It is tempting to speculate that the relocalisation of Setdb1 to the cytoplasm could also lead to methylation of non-histone proteins in the cytoplasm. Concomitantly, the genomic relocalisation of the KMT could facilitate subsequent demethylation of H3K9 and/or non-histone proteins at specific nuclear loci. We noticed a decrease in total Setdb1 protein level in late differentiation times, with a shift in the Setdb1 migration profile compared with proliferating conditions, suggesting that Setdb1 could be ubiquitinated and subsequently degraded by the proteasome. To proof this further investigation will be required.

Our work shows genomic release of Setdb1 from only a subset of target genes. This could offer a fine-tuning mechanism for subsequent gene activation. Further work will reveal what determines the Setdb1 pool for removal from selected genes and export from the nucleus. It would be interesting to test if certain Wnt-induced post-translational modifications of Setdb1 itself generate a 'code' for its transport to the cytoplasm. For example, specific phosphorylation of HDAC4 and HDAC5 in cardiomyocytes and skeletal myoblasts, respectively, is required for their nuclear export [70, 71]. Interestingly, a recent paper by Singh *et al.* [72] showed that the co-repressor KAP1 (Krüppel-like associated box-associated protein 1, also called Trim28), a known Setdb1 partner [16], represses MyoD and MEF2 in myoblasts and that upon differentiation, MSK1-mediated phosphorylation of KAP1 releases it from MyoD/MEF2 allowing their activation. It is thus possible that Setdb1 could be part of the same complexes and undergo comparable regulation. However, Singh *et al.* detected Trim28 but not Setdb1 in the MyoD complex and Song *et al.* [66] failed to detect any interaction between Setdb1 and MyoD in co-immunoprecipitation assays. In addition, only 30/150 genes upregulated upon Trim28 knockdown where also upregulated (>2000 genes) upon Setdb1 KD (not shown). Thus, it is unlikely that Trim28 and Setdb1 would have the same mechanism of action in myoblasts.

While it was demonstrated in colon cancer cells that the canonical Wnt pathway recruits Setdb1 at Wnt target gene promoters for transcriptional repression [73], we uncovered a novel mechanism of Wnt-mediated gene activation by subcellular redistribution of Setdb1. This further provides evidence for selective mechanisms possibly based on targets and tissues. To understand what specifies the subset of Setdb1 target genes regulated in a Wnt-dependent manner and during differentiation, it would be interesting to study the chromatin landscape at these specific promoters. A particular chromatin signature could influence Setdb1-binding affinity and facilitate its release from certain loci, while it could recruit Setdb1 to other loci through Wnt3a.

Among the identified Setdb1 genomic targets, *Ankrd1* appeared of particular interest due to its involvement in myogenesis [41] and its Wnt/β-Catenin-dependent induction in mammary tumours [54]. Here we demonstrated a crucial role for Ankrd1 in MuSC proliferation and myoblast terminal differentiation. The underlying mechanism of Ankrd1 function is unknown; however, it was recently shown to act as transcriptional co-activator of p53 [74] or repressor of the androgen receptor [75]. It remains to be demonstrated whether Ankrd1 is involved in a complex with myogenic transcription factors and whether it regulates gene expression during muscle terminal differentiation.

The effect of Ankrd1 overexpression on terminal differentiation was already studied both *in vitro* in myoblasts [75] and *in vivo* [76]. Overexpression of Ankrd1 *in vivo* in mice did not affect weight, neither the number nor diameter of fibres, but induced reduction of slow-twitch fibre type [76]. This is in agreement with an *Ankrd1* gene knockout study, which suggested that Ankrd1 plays a role in fibre typing [77]. However, it is important to note that the study by Barash *et al.* did not describe any skeletal muscle injury experiments. Thus, this work does not rule out any role for Ankrd1 during adult regenerative myogenesis (which is mimicked by muscle stem cells on single myofibres in our hands). Importantly, Ankrd1 is not expressed in healthy uninjured skeletal muscle tissue, but strongly induced both during myofibre regeneration following injury and in pathological contexts [42]. It is thus likely that Ankrd1 regulates MuSCs function *in vivo* during muscle tissue response to injury as suggested by both its expression profile and our *ex vivo* single myofibre experiments. Further, it was well documented that, in many cases, mutations that affect adult (regenerative) myogenesis do not affect muscle formation and uninjured muscle physiology. For example, *FGF6*-null mice have normal skeletal muscle formation but show a severe regeneration defect with fibrosis and myotube degeneration [78]. Likewise, the *Notch3*-deficient mice have a perfectly normal musculature, but present a remarkable overgrowth of muscle mass only when they suffered repetitive muscle injuries [79].

Interestingly, our results showed that Setdb1 knockdown not only affects Ankrd1 expression levels but also Ankrd1-related genes, such as *Ttn* and *Mmp13*. Titin is a Ankrd1 partner within the mechanosensory signalling complex in the sarcomere [41], and Mmp13 expression is negatively regulated by Ankrd1 as a transcriptional co-repressor [61]. MMP13 being itself recently shown to play an important role in the muscle repair process, and blockade of MMP13 expression in myoblasts resulted in a delay in differentiation [80]. These data further shed light on the functional link between Setdb1 and the 'Ankrd1 pathway'.

In conclusion, we demonstrated for the first time the subcellular relocalisation of an essential epigenetic modifier by the canonical Wnt signalling as a mode of gene regulation. It will be interesting to study whether Wnt-dependent relocalisation of Setdb1 is a general mechanism in proliferating and differentiating cells. If the localisation of an epigenetic modifier, rather than its changed catalytic activity or expression, plays a crucial role in cell fate, targeting its subcellular localisation could offer a new pharmacological and therapeutic approach for multiple pathologies.

## Materials and Methods

### Cell lines and culture

C2C12 myoblast cell line was grown in high-glucose Dulbecco’s modified Eagle’s medium (DMEM; Invitrogen, Courtaboeuf, France) with 1% penicillin/streptomycin, sodium pyruvate and 20% fetal calf serum (FCS, PAA). Differentiation was initiated at 80–90% confluence in high-glucose DMEM containing penicillin/streptomycin, 1% sodium pyruvate (PAA) and 2% horse serum (Gibco, Courtaboeuf, France), for the indicated period of time.

Proliferating primary myoblasts were cultured on rat-tail collagen (Gibco)-coated dishes in Ham’s F-10 medium (Life Technologies, Villebon sur Yvette, France), 20% FCS, 2 ng l^−1^ basic fibroblast growth factors (R&D Systems, Lille, France), 1% penicillin/streptomycin, as described previously [81]. For differentiation, primary myoblasts were kept in high-glucose DMEM containing 1% penicillin/streptomycin, 1% sodium pyruvate and 4% horse serum.

C2C12 myoblasts stably expressing double-tagged Flag-HA-Setdb1 wild-type protein or the H1224K catalytically inactive protein were established as previously described (Joliot, 2014). As control C2C12 myoblasts were transduced with the empty pREV vector.

When indicated, HeLa cells, C2C12 and primary myoblasts were treated with 10 ng ml^−1^ Leptomycin B solution from *Streptomyces* sp for 10 and18 h, respectively (LMB; Sigma; #L2913), 50 ng ml^−1^ Wnt3a (#1324-WN; R&D systems) and 20 μm IWP2 (#3533; R&D systems) both for 24 h.

Wild-type and *Eset* knockout mESCs were cultured as described in [34].

### Antibodies (Ab)

Rabbit polyclonal Setdb1 antibodies (#sc-66884x, 1:200 in IF; #sc-66884 1:2 000 in WB), 5 μg in ChIP, mouse monoclonal CARP antibody (alias Ankrd1, #sc-365056, 1:500 in WB), goat polyclonal CTCF antibody (#sc-15914, 1:500, WB), rabbit polyclonal MyoD antibody (#sc-304, 1:100 in IF), rabbit polyclonal Myogenin antibody (#sc-576, 1:100 in IF), mouse monoclonal Pax7 antibody (#sc-81648, 1:100 in IF) and the mouse monoclonal Myogenin (#sc-576, 1:1000 in WB) were from Santa Cruz (Dallas, TX, USA). Mouse monoclonal active β-Catenin antibody (#05-665, 1:1 000 in WB) was from Millipore (Molsheim, France). The mouse monoclonal α-Tubulin antibody (# T9026, 1:2 000 in WB), the mouse monoclonal MyHC antibody (#M4276, 1: 1 000 in WB), the mouse monoclonal β-Actin antibody (#A5441, 1: 10 000 in WB) and the mouse monoclonal Vinculin antibody (#V9131, 1:10 000 in WB) were from Sigma Aldrich (Saint Quentin Fallavier, France). Rat monoclonal Hsc70 antibody (#ADI-SPA-815, 1:1000 in WB) was from Enzo Life Sciences (Villeurbane, France). The rabbit monoclonal non-phospho β-Catenin antibody (#8814, 1:100, IF) was purchased from Cell Signalling Technology (Saint Quentin en Yvelines, France). The rabbit polyclonal antibodies H3K4me1 (#C15410037, 3 μg, ChIP), H3K9ac (#C15410004, 3 μg, ChIP) and H3K27ac (#C15410174, 3 μg, ChIP) were purchased from Diagenode (Seraing, Belgium). The Ckm antibody (1:100 in IF) was a kind gift from Hidenori ITO (Aichi, Japan). Secondary antibodies used for IF (# 711-586-152, #715-096-150, 1:800) were from Jackson Immunoresearch (Suffolk, UK). Secondary antibodies used for WB were purchased from Thermo Fisher Scientific (Courtaboeuf, France) (anti-rabbit IgG, #31460, 1:25 000) and Sigma Aldrich (anti-mouse IgG, #A2304, 1:25 000).

### Animal experimentation

Experimental animal protocols were performed in accordance with the guidelines of the French Veterinary Department and approved by the University Paris Descartes Ethical Committee for Animal Experimentation. All experiments were performed in 8-week-old mice. Our animals are on C57Bl6N genetic background.

### Single myofibre assay

Single myofibres were isolated from the extensor digitorum longus muscle by Collagenase type I digestion and gentle trituration, as previously described [29]. Isolated myofibres were cultured in suspension for up to 3 days in six-well plates coated with horse serum to prevent fibre attachment. Fibres were incubated in plating medium consisting of 15% FCS and 1% Chick Embryo Extract (Accurate Chemicals, Courtaboeuf, France) in DMEM. For each fibre assay a minimum of three independent experiments was performed. Each experiment corresponded to one mouse. Between 25 and 30 fibres were counted per experiment. For immunostaining, single myofibres are fixed in 2% PFA in phosphate-buffered saline (PBS), washed three times in PBS and stored at 4 °C for 1 h in blocking solution consisting of 5% goat serum, 0.5% bovine serum albumin and 0.2% Triton-X in PBS (all from Sigma). This is followed by an overnight incubation at 4 °C with primary antibodies diluted in blocking solution. After 3 washes in PBS the fibres are incubated for 1 h at room temperature (RT) with secondary antibodies conjugated to a fluorescent dye (Alexa Fluor 488 or 568; Invitrogen-Molecular Probes (Courtaboeuf, France)) in PBS. The staining is completed with three washes with PBS and incubation in Hoechst solution to label cells nuclei. Fibres are mounted on glass slide in Dako Mounting medium.

### Primary myoblast preparation

For isolation of primary myoblasts, skeletal muscles of the hindlimbs from 6- to 8-week-old mice were dissected with care to take off as much fat and connective tissue as possible [82]. Muscles were transferred to a sterile Petri dish on ice, mulched into a smooth pulp and incubated in Collagenase B/Dispase II/CaCl_2_ solution (Roche, Boulogne-Billancrout, France, respectively 1.5 U ml^−1^; 2.4 U ml^−1^ and 2 M, in DMEM). Following a 15- min incubation at 37 °C, the muscle pulp was triturated with a heat polished glass Pasteur pipettes. This trituration/incubation step was repeated once. Tissue digestion was stopped with addition of FCS, cells were filtered, washed twice with phosphate-buffered saline and resuspended in growth medium consisting of Ham’s F-10 medium supplemented with 20% fetal bovine serum and 2.5 ng μl^−1^ of basic fibroblast growth factor. After 2 h of pre-plating in a non-coated 10 cm plate, the medium was transferred onto collagen-coated Petri dishes. Cultures were maintained in growth medium until cells reached 80% confluence. To enrich cell cultures in myoblasts and eliminate contaminating fibroblasts, cell dishes were tapped. Owing to differential adhesion, this led only myoblasts to be detached and re-plated onto new plates.

### siRNA transfection

C2C12 cells were transfected at 80–90% confluence using HiPerFect transfection reagent (Qiagen, Les Ulis, France) to achieve a final siRNA concentration of 33 nm. Differentiation of cells was started immediately after first transfection as described above. For siMyogenin experiments, cells were transfected with a pool of two siRNA and processed 24 h after transfection. For siSetdb1 experiments a second transfection was performed 24 h after the first transfection. Cells were incubated 72 h post first transfection before being processed for analysis. Single extensor digitorum longus myofibers were transfected 2 h after isolation using Lipofectamine 2000 reagent (Life Technologies) to obtain a final concentration of 33 nm. Fibres were then transferred in fresh media 6 h post-transfection, and fixed 72 h post-transfection. RNA interference was conducted with at least two independent siRNAs (Sigma), which gave similar results. Specific sequence information are described below.

### Classification of Setdb1 localisation

To characterise Setdb1 subcellular localisation in C2C12 myoblasts (Figures 4 and 5 and Supplementary Figures S4 and S5), confocal microscopy images were analysed. Setdb1 subcellular distribution was classified in three categories. Setdb1 localisation was considered as *mainly cytoplasmic*, when the staining appeared stronger in the cytoplasm than in the nucleus. The two compartments were clearly distinguishable by Setdb1 staining only. For representative images see Supplementary Figure S4A (upper). Setdb1 localisation was considered as *homogeneous* if distribution appeared similar throughout the cell. In this case, a clear discrimination between cytoplasm and nucleus by Setdb1 staining only was not possible. For representative images see Supplementary Figure S4A (middle). Setdb1 localisation was referred to as *mainly nuclear*, if distribution was concentrated in the nucleus. A discrimination of the compartments by Setdb1 staining only was feasible. For representative images refer to Supplementary Figure S4A (lower).

### Western blotting (WB)

Cells were lysed in RIPA buffer supplemented with phosphatase and protease inhibitors (Sigma). Whole-cell extracts were separated on a pre-cast NuPage 4–12% acrylamide gradient sodium dodecyl sulphate polyacrylamide gel electrophoresis gel (Invitrogen) and transferred to a nitrocellulose membrane (Thermo Fisher Scientific) in Tris-glycine transfer buffer. Membranes were blocked with 10% milk and incubated overnight at 4 °C with the primary Abs. Membranes were incubated with the appropriate secondary Abs coupled with horseradish peroxydase for 1 h at RT. Membranes were revealed using SuperSignal West Dura Extended Duration Substrate (Thermo Fisher Scientific) and images were taken with ChemiSmart 5000 system (Vilber Lourmat, Collégien, France).

### Reverse transcription and quantitative real-time PCR (RT-qPCR)

Total RNA was isolated using the RNeasy mini kit from Qiagen. DNase (Qiagen) treatment was included to remove residual DNA. cDNA was generated by Taq-Man Reverse Transcription Kit (Applied Biosystems) or the High Capacity cDNA RT kit (Life Technologies). qPCR was performed with SYBR Green 2x master mix (Applied Biosystems, Courtaboeuf, France) using the 7300 ABI Prism Real-time PCR machine (Applied Biosystems) or the LC480 Real-time PCR machine (Roche). Relative mRNA expressions of genes of interest were calculated with the ΔΔ*C*_T_ method, using cyclophilin A (CycloA) and/or TATA-box-binding protein (Tbp) as house keeping gene. See below for primer sequences.

### IF and microscopic analysis

Cells were grown on glass coverslips, which had been coated with collagen type I (Sigma) for 1 h at 37 °C and rinsed with PBS. C2C12 cells were fixed with 4% formaldehyde (FA; Sigma), incubated with 0.1 m glycine for 5 min to quench FA, permeabilised with 0.2% Triton-X-100 and blocked with PBS containing 2% donkey serum (Jackson Immunoresearch) for 1 h at RT. Primary and secondary Abs were diluted in PBS containing 2% donkey serum and 0.2% Tween and incubated overnight at 4C° or 1 h at RT, respectively. DNA was stained with 1 μg ml^−1^ DAPI (Sigma). Coverslips were mounted with Fluorescence Mounting Media (Dako). Primary myoblasts and fibres were processed in the same way; except blocking was done with 5% heat inactivated serum in PBS and DNA were counterstained with Hoechst (Life Technologies).

Microscopy was performed using the Axioplan 2 (Zeiss). Images were taken with the Cool Snap fx camera (Photometrics) and analysed with Metamorph software. Confocal microscopy was performed using the LM710 microscope (Zeiss, Marly le Roi, France). Confocal images were analysed with ZEN 2011 software. Light microscopy was performed using the Leica DMIL LED microscope. All images were processed with ImageJ software (http://rsbweb.nih.gov/ij/) to identical contrast and brightness.

### TUNEL assay

Apoptotic cells were stained by performing the TUNEL reaction, using the *In situ* Cell death detection kit (#11684795910; Roche). The procedure was according to the manufacturers instructions.

### ChIP and qPCR

FA was added to culture medium to a final concentration of 1%. Crosslinking was allowed to proceed for 10 min at RT and stopped by addition of glycine at a final concentration of 0.125 m. Fixed cells were washed and harvested with PBS. Cells were lysed in Buffer 1 (50 mm Hepes/KOH pH 7.5; 140 mm NaCl; 1 mm EDTA; 10% Glycerol; 0.5% NP-40; 0.25% Triton). The resulting pellet was resuspended in Buffer 2 (200 mm NaCl; 1 mm EDTA; 0.5 mm EGTA; 10 mm Tris pH 8). Each resuspension step was followed by 10 min incubation at 4 °C. Nuclei were then pelleted by centrifugation, resuspended in Buffer 3 (50 mm Tris pH 8; 0.1% SDS; 1% NP-40; 0.1% Na-Deoxycholate; 10 mm EDTA; 150 mm NaCl). All buffers were supplemented with phosphatase and protease inhibitors prior to usage. Samples were sonicated with Bioruptor Power-up (Diagenode) yielding genomic DNA fragments with a bulk size of 150–300 bp. Chromatin was precleared with Protein A/G ultralink beads (#53133; Thermo Fisher Scientific) for 1 h at 4 °C and immunoprecipitation with the specific Abs overnight at 4 °C. Immunocomplexes were recovered by adding protein A/G ultralink beads, preblocked with bovine serum albumin and incubated for 2 h at RT. Beads were washed twice with low salt buffer (0.1% SDS; 1% Triton; 2 mm EDTA; 20 mm Tris pH 8; 150 mm NaCl), twice with high salt buffer (0.1% SDS; 1% Triton; 2 mm EDTA; 20 mm Tris pH 8; 500 mm NaCl), once with LiCl buffer (10 mm Tris pH 8.0; 1% Na-deoxycholate; 1% NP-40, 250 mm LiCl; 1 mm EDTA) and twice with TE buffer+50 mm NaCl. All buffers were supplemented with phosphatase and protease inhibitors. Chromatin was eluted with Elution buffer (50 mm Tris, pH 8; 1 mm EDTA; 1% SDS; 0.2 m NaCl) at 65 °C and crosslinking was reversed overnight at 65 °C using Proteinase K (Sigma). The eluted material was phenol/chloroform-extracted and ethanol-precipitated. DNA was resuspended in water and qPCR was performed as described above. ChIP-qPCR results are represented as percentage (%) of IP/input signal (% input). Owing to different chromatin compaction during proliferation and differentiation, we additionally normalised specific enrichments (% input) of Setdb1 and H3K9me3 to the corresponding IgGs, which served as negative control. ChIP-qPCR primer sequences are listed below.

### ChIP-sequencing (seq)

SETDB1 ChIP was performed as described above. Five to 15 ng of ChIPed DNA or whole-cell extract (Input) were prepared for sequencing on Illumina Hiseq 2000. We used the library kit (Truseq DNA sample prep kit V2; Illumina, Paris, France) according to the manufacturers’ instructions with the following modifications: DNA fragments were repaired to blunt ends, purified with magnetic beads (Agencourt AMPure XP; Beckman Coulter, Villepinte, France) and a step of A-tailing was performed before ligating to Illumina adapters. Two steps of DNA purification on magnetic beads were carried out to eliminate non-ligated adaptors and then amplified with 15 PCR cycles. To remove non-ligated left adapters and large DNA fragments, DNA libraries were selected on E-Gel (2% SizeSelect; Invitrogen) to obtain 280–330 bp DNA fragments (including 130 bp of adapters). To ensure high quality of the library, samples were tested using the DNA high-sensitivity chip (Agilent Technologies, Les Ulis, France) and for positive target enrichment by qPCR. Samples were then quantified by qPCR and PicoGreen (Qubit 2.0 Fluorometer; Invitrogen). Libraries were pooled thanks to various adaptors and qPCR relative measurements. The cluster amplification and following sequencing steps were performed following the Illumina standard protocol.

### Statistical parameters for ChIP-seq analysis

Sequenced reads were demultiplexed to attribute each read to a DNA sample and then aligned to reference mouse genome *mm9* with bowtie (*-n 2 -e 70 -l 50 --maxbts 125 -k 1 -m 1*). After PCR duplicates were removed, Setdb1 and H3K9me3 enrichments were analysed over their respective control (Input DNA) by the software MACS 1.4 as previously described [83]. The *P*-value threshold was 10e-3, and the shift size 50 pb (-shiftsize 50 -no model). For H3K9me3 ChIP-seq an additional peak caller was used: SICER v 0.0.1 (*e*-value <0.01) with W200 and G1000 parameters. Setdb1 ChIP-seq peaks were determined on Setdb1 ChIP-seq and only binding sites with *P*-values <10e-5, FDR<1% and fourfold enrichment over the control were retained. For genome annotation we used Homer software v4.7 [84]. For bound genes, we used GREAT software (v3.0). Plots were generated with *ngsplot* package [85]. Genomatix software was used for Gene Ontology analyses. Hypergeometric test has been performed in R.

### RNA purification and library construction for RNA-seq

RNA was isolated as described above. Polyadenylated messenger RNA was purified from 1 μg RNA and the library was generated using the TruSeq stranded mRNA library prep Kit, set A and B (Illumina; #RS-122-2101 and #RS-122-2102). The obtained directional libraries were controlled by Bioanalyzer DNA1000 Chips (Agilent; # 5067-1504) and quantified by spectrofluorimetry (QuantiT High-Sensitivity DNA Assay Kit, #Q33120; Invitrogen).

### Sequencing and bioinformatic analysis of RNA-seq

Sequencing was performed on a HiSeq 2500 (Illumina) in a 51 bases single read using a HiSeq SR Cluster kit v4 cBot HS (Illumina; # GD-401-4001) and a HiSeq SBS kit v4 HS 50 cycles (Illumina; # FC-401-4002). Sequences were demultiplexed using the Illumina pipeline (Gerald, included in CASAVA version 1.8) giving FASTQ formatted reads. Those reads were cleaned from adapter sequences and sequences of low quality using an in-house programme (https://github.com/baj12/clean_ngs). Sequences with a minimum length of 25 nucleotides were considered for further analysis. Tophat (version 1.4.1.1, default parameters) was used to align to the reference genome (mm9). HTseq-count (parameters: -m intersection-nonempty, -s yes, -t exon) was used for counting genes [86, 87]. Dseq2 package [88] was used for statistical analysis.

### Statistical analysis

All values are presented as mean±s.d. (s.e.m.). For statistical significance Student’s paired *t*-test was applied. For Gene Ontology analysis Fisher exact test was applied. *P*-values less than 0.05 (*) were considered significant. *P*-values less than 0.01 are marked as [Table tbl1].

### siRNA sequences

[Table tbl2]

### Primer sequencesRT-qPCR

qPCR primers (ChIP)

[Table tbl3]

### Accession Numbers

Canonical Wnt signalling regulates nuclear export of Setdb1 during skeletal muscle terminal differentiation: PONTIS Julien, 2016, http://www.ncbi.nlm.nih.gov/geo/query/acc.cgi?token=edkvkumqdzwbdgp&acc=GSE70070

## Figures and Tables

**Table 1 tbl1:** 

*Target*	*Forward*	*Reverse*
Mouse Setdb1	GCGCAGAGUUAACCGCAAAUU	UUUGCGGUUAACUCUGCGCUU
Mouse Ankrd1	CAGAUGUCCUGAAACUGUU	AACAGUUUCAGGACAUCUG
Mouse *Myogenin* Nr.1	CAGACGAAACCAUGCCCAA[dT][dT]	UUGGGCAUGGUUUCGUCUG[dT][dT]
Mouse *Myogenin* Nr. 2	CCAGUACAUUGAGCGCCUA[dT][dT]	UAGGCGCUCAAUGUACUGG[dT][dT]

**Table 2 tbl2:** 

*Target*	*Forward*	*Reverse*
Mouse *Setdb1*	GCAATGGAGAAGAAGCAAGG	ATAGGCTGTAGGGGCTCCAT
Mouse *Ankrd1*	CGGACCTCAAGGTCAAGAAC	GCTCTTCTGTTGGGAAATGC
Mouse *Cmk*	CACCATGCCGTTCGGCAACA	GGTTGTCCACCCCAGTCT
Mouse *Ccnd1*	GCGTACCCTGACACCAATCT	CTCTTCGCACTTCTGCTCCT
Mouse *Axin2*	AAGAGAAGCGACCCAGTCAA	CTGCGATGCATCTCTCTCTG
Mouse *CycloA*	GTCAACCCCACCGTGTTCTT	GTCAACCCCACCGTGTTCTT
Mouse *Tbp*	ACGCGACCGCAGAAACCTAGC	TGCGTCAGGCGTTCGGTGGAT

**Table 3 tbl3:** 

*Target*	*Forward*	*Reverse*
Mouse *Ankrd1*	GGTAAGGTAAAGGTTGGAGATCTGAT	GGCTTGTCTGGGATTTCTTCTGT
Mouse Atxn10	CTGGTTGATGGACCCAAAGAA	CTGGTTGATGGACCCAAAGAA
