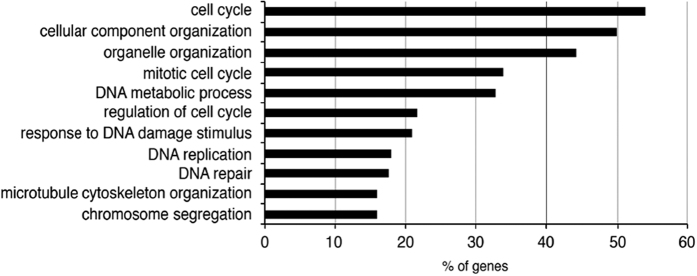# Canonical Wnt signalling regulates nuclear export of Setdb1 during skeletal muscle terminal differentiation

**DOI:** 10.1038/celldisc.2016.43

**Published:** 2016-11-15

**Authors:** Sophie Beyer, Julien Pontis, Elija Schirwis, Valentine Battisti, Anja Rudolf, Fabien Le Grand, Slimane Ait-Si-Ali

**Affiliations:** 1 Centre National de la Recherche Scientifique CNRS-Université Paris Diderot, Sorbonne Paris Cité, Epigenetics and Cell Fate UMR7216, Paris, France; 2 Institut Cochin, Université Paris-Descartes, Centre National de la Recherche Scientifique (CNRS) UMR8104, Paris, France; 3 Institut National de la Santé et de la Recherche Médicale (INSERM) U1016, Paris, France

**Correction to:** *Cell Discovery* (2016) **2**, 16037; doi:10.1038/celldisc.2016.37; Published 18 October 2016

In the initial published version of this article, an error was made in Figure 6c, where the titles of *x* axis and *y* axis were left out. The corrected Figure 6c is now attached in this corrigendum. All figures and text description other than this remain the same as initially published. The mistake does not affect the results and the conclusion of the paper. We sincerely apologize for any inconvenience that may have been caused by this error.

## Figures and Tables

**Figure 6:**