# Supplementary material for: Canonical Wnt signalling regulates nuclear export of Setdb1 during skeletal muscle terminal differentiation
Source: Cell Discov. 2016 Oct 18;2:16037–. doi: 10.1038/celldisc.2016.37 (PMC5067623; doi:10.1038/celldisc.2016.37)
Supplement: Supplementary Information [file celldisc201637-s1.pdf]

Figure S1, Beyer et al.

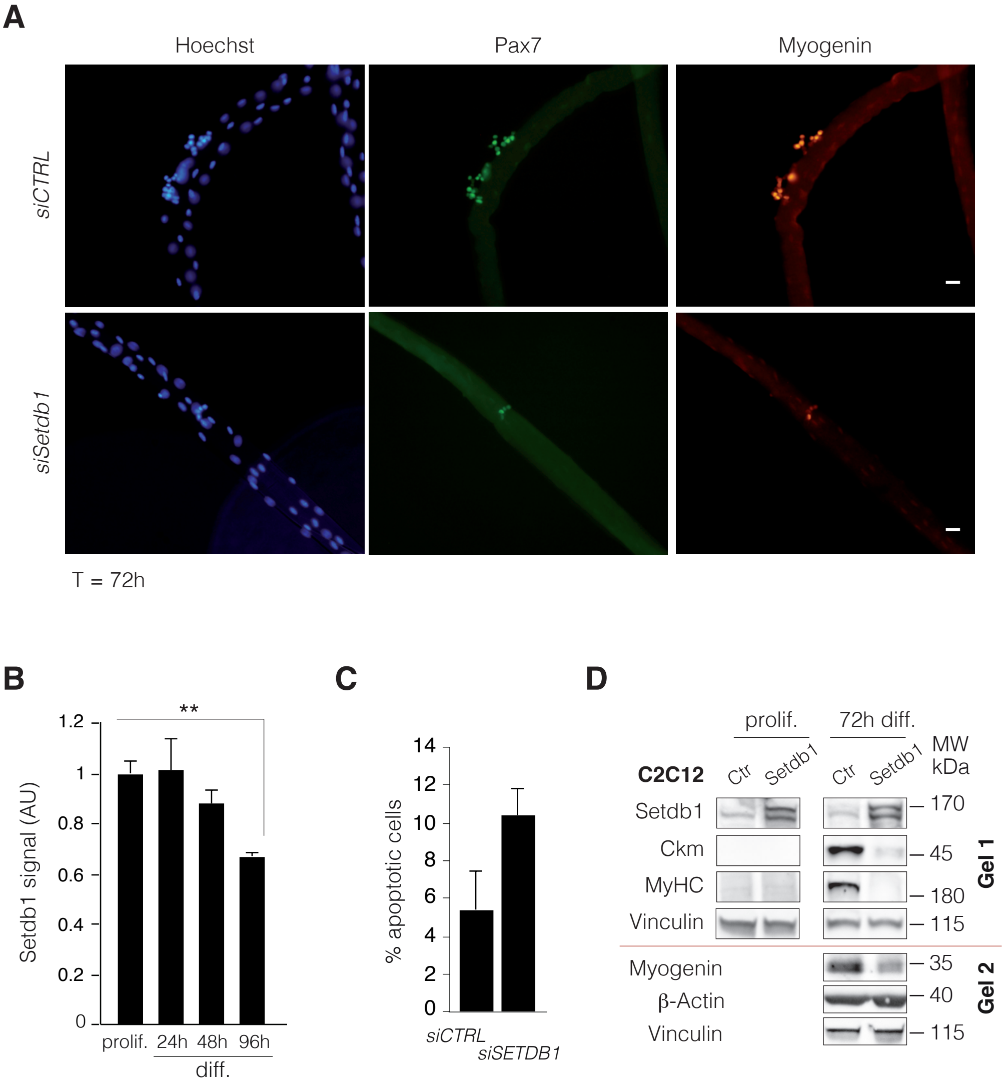

Figure S1: Setdb1 is required for accurate skeletal muscle stem cell proliferation.

(A) Setdb1 depletion inhibits MuSC proliferation. EDL single myofibres were cultured for 72 h following transfection with control (siCTRL) or Setdb1 siRNA (siSetdb1). For detection of proliferating and differentiating MuSCs indirect IF was performed to detect Pax7 (green) and Myogenin (red), respectively. DNA was labelled with Hoechst (blue). Representative myogenic cell clusters are shown. Scale bar = 5 µm. This figure supplements Figure 1E.
(B) Setdb1 protein decreases with progressive differentiation. Quantification of WB for Setdb1 as described in Figure 1G. The Setdb1 signal intensity was measured with ImageJ software. Every signal was normalized to the corresponding loading control.
(C) Proliferating C2C12 myoblasts were transfected and differentiated for 72 h as described in Figure 1H. TUNEL assay was performed to detect apoptotic cells, as in Figure 3F. Data are presented as mean +/- SEM of three independent experiments. For significance Student paired t-test was applied. The difference between the two conditions was not statistically significant.
(D) Overexpression of Setdb1 inhibits expression of muscle differentiation markers. WB analysis of Setdb1, MyHC, Ckm and Myogenin was performed in whole cell extracts from C2C12 cells stably overexpressing Setdb1 or an empty expression vector (Ctr). Cells were proliferating (prolif.) or differentiated (diff.) for 72 h. Vinculin and β-Actin; loading controls. Note that the shown lanes were cut from the same gels (1 or 2) and have strictly the same exposure time.

For A and D: Images are representative of a minimum of three independent experiments.
*For B and C: Data are presented as mean +/- SEM of three independent experiments. For significance Student paired t-test was applied. **: p-values less than 0.01 and are considered significant.*

Figure S2, Beyer et al.

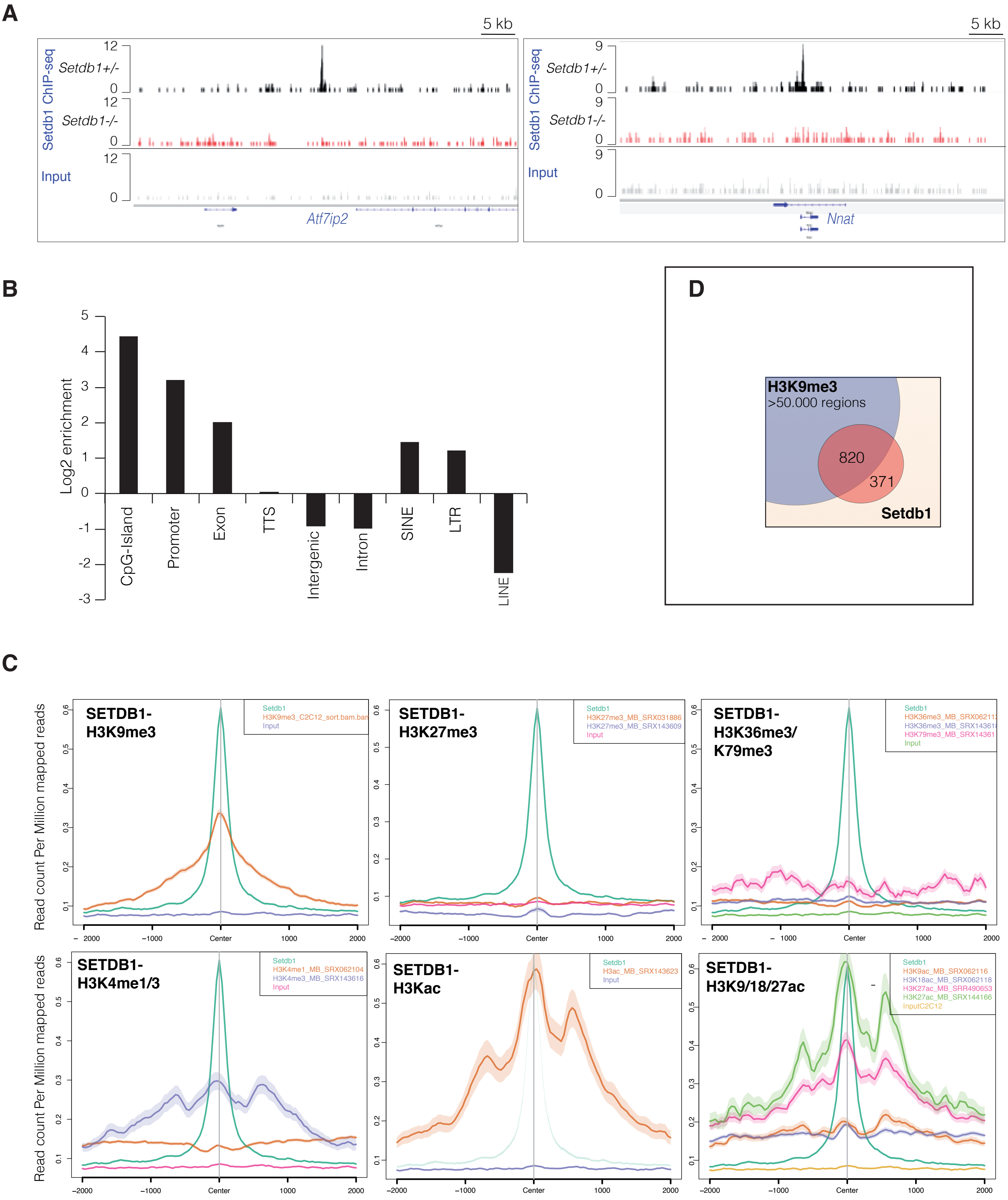

Figure S2: Comparison of genomic distribution of Setdb1 and histone marks in proliferating myoblasts and ChIP-seq specificity controls

(**A**) Genome Browser presentation of Setdb1 binding at the promoter of *Atf7ip2* and *Nnat,* analysed by ChIP-seq in mouse embryonic stem cells (mESC) heterozygous (*Setdb1+/-*) or knockout (*Setdb1-/-*) for *Setdb1*.

(**B**) Presentation of genomic elements bound by Setdb1 in C2C12. Enrichments were analysed by ChIP-seq. This figure supplements Figure 2A.

(**C**) Comparison of enrichments between various histone modifications and Setdb1. Presented are ChIP-seq analyses in proliferating myoblasts (MB). Setdb1/H3K9me3 enrichment and input were analysed in proliferating C2C12 myoblasts. H3K27me3 data were re-analysed from [1] and H3K27ac data from [2]. Remaining histone modifications were re-analysed from [3]. The scale is +/- 2kb from Setdb1 peak summits and the enrichment corresponds to the normalised number of reads.

(**D**) Venn Diagram showing 820 genes commonly enriched by Setdb1 and H3K9me3 in proliferating C2C12 myoblasts. Data were analysed by ChIP-seq: for H3K9me3 enrichment, 2 peak callers were used and merged (MACS1.4, p-value <0.001; and SICER v0.0.1, e-value < 0.01).

References

1 Mousavi K, Zare H, Wang AH, Sartorelli V. Polycomb protein Ezh1 promotes RNA polymerase II elongation. Molecular cell 2012; **45**:255-262.

2 Blum R, Vethantham V, Bowman C, Rudnicki M, Dynlacht BD. Genome-wide identification of enhancers in skeletal muscle: the role of MyoD1. Genes Dev 2012; **26**:2763-2779.

3 Asp P, Blum R, Vethantham V et al. Genome-wide remodeling of the epigenetic landscape during myogenic differentiation. Proceedings of the National Academy of Sciences of the United States of America 2011; **108**:E149-158.

Figure S3, Beyer et al.

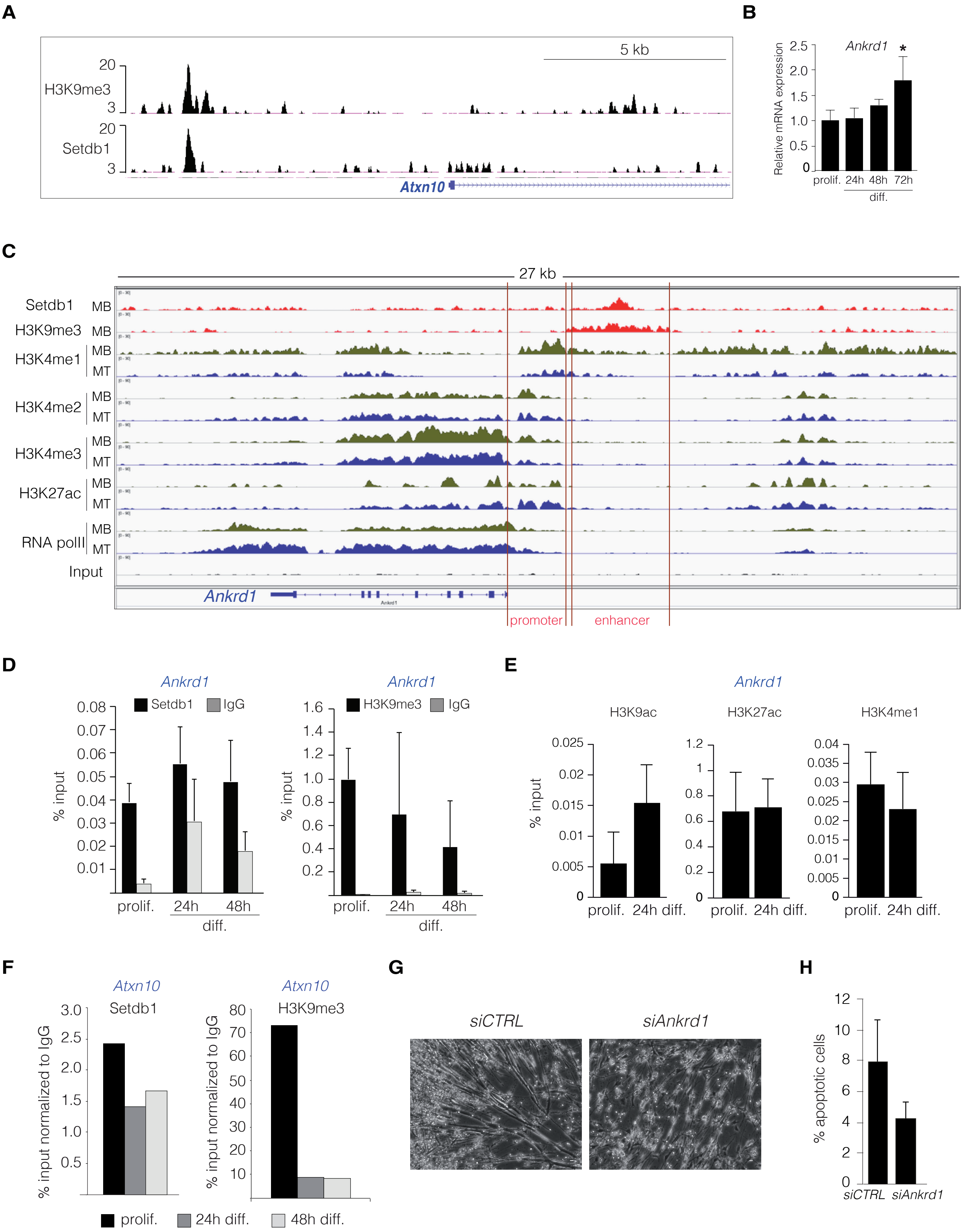

Figure S3: A subset of Setdb1 target genes are bound differently during terminal differentiation in myoblasts.

(**A**) Genome Browser presentation of Setdb1 and H3K9me3 binding profiles at *Atxn10* promoter in proliferating C2C12 myoblasts analysed by ChIP-seq.

(**B**) Relative mRNA expression analysis of *Ankrd1* increases during differentiation in primary myoblasts. Cells were proliferating (prolif.) or differentiated (diff.) for the indicated time (24, 48 or 72 h). This figure supplements Figure 2H.

(**C**) ChIP-seq representation of Setdb1 and H3K9me3 bindings (both red) at the *Ankrd1* gene in proliferating C2C12 myoblasts. Binding profiles are compared with published data for H3K4me1, H3K4me2, H3K4me3, and RNA pol II by Asp P *et al*., 2011 [1]. H3K27ac binding data are published by Blum R. *et al*., 2012 [2]. H3K9me3 binding data are from [3]. Binding was analysed in myoblasts (MB) in green and myotubes (MT) in blue.

(**D**) Setdb1 (left) and H3K9me3 (right) enrichments at the *Ankrd1* enhancer. C2C12 cells were proliferating (prolif.) or differentiating (diff.) for 24 h or 48 h. Binding was analysed by ChIP-qPCR. Results are presented as immunoprecipitated DNA compared to input DNA (% input). IgG served as a negative control. *Presented data are mean +/- SEM of a minimum of three independent experiments.* This figure supplements Figure 2I, J.

(**E**) H3K9ac, H3K27ac and H3K4me1 binding was analysed by ChIP-qPCR at the *Ankrd1* enhancer. C2C12 cells were proliferating (prolif.) or differentiating for 24 h (24h diff.). Results are presented as immunoprecipitated compared to input DNA (% input). *Presented data are mean +/- SEM of a minimum of three independent experiments.*

(**F**) Setdb1 (left) and H3K9me3 (right) enrichments at the *Atxn10* promoter were analysed by ChIP and qPCR. C2C12 myoblasts were proliferating (prolif.) or differentiating (diff.) for 24 h or 48 h. Results are presented as % input and normalised to IgG (negative control). Results are from one experiment as representative of a minimum of three independent experiments.

(**G**) Phase contrast images of C2C12 myoblasts after Ankrd1 knockdown. 80-90% confluent cells were transfected with control siRNA (*siCTRL*) or Ankrd1 siRNA (*siAnkrd1*). Differentiation was started simultaneously for 72 h. Scale bar = 10 μm.

(H) Proliferating C2C12 myoblasts were transfected and differentiated as described in Fig S1C. Apoptotic cells were stained by performing the *TdT-mediated dUTP-biotin nick end labeling* (TUNEL) reaction. Cells containing a signal inside the nucleus were considered as apoptotic. A minimum of 400 cells was counted.

References

1 Asp P, Blum R, Vethantham V *et al.* Genome-wide remodeling of the epigenetic landscape during myogenic differentiation. *Proceedings of the National Academy of Sciences of the United States of America* 2011; **108**:E149-158.

2 Blum R, Vethantham V, Bowman C, Rudnicki M, Dynlacht BD. Genome-wide identification of enhancers in skeletal muscle: the role of MyoD1. *Genes Dev* 2012; **26**:2763-2779.

3 Mousavi K, Zare H, Wang AH, Sartorelli V. Polycomb protein Ezh1 promotes RNA polymerase II elongation. *Molecular cell* 2012; **45**:255-262.

Figure S4, Beyer et al.

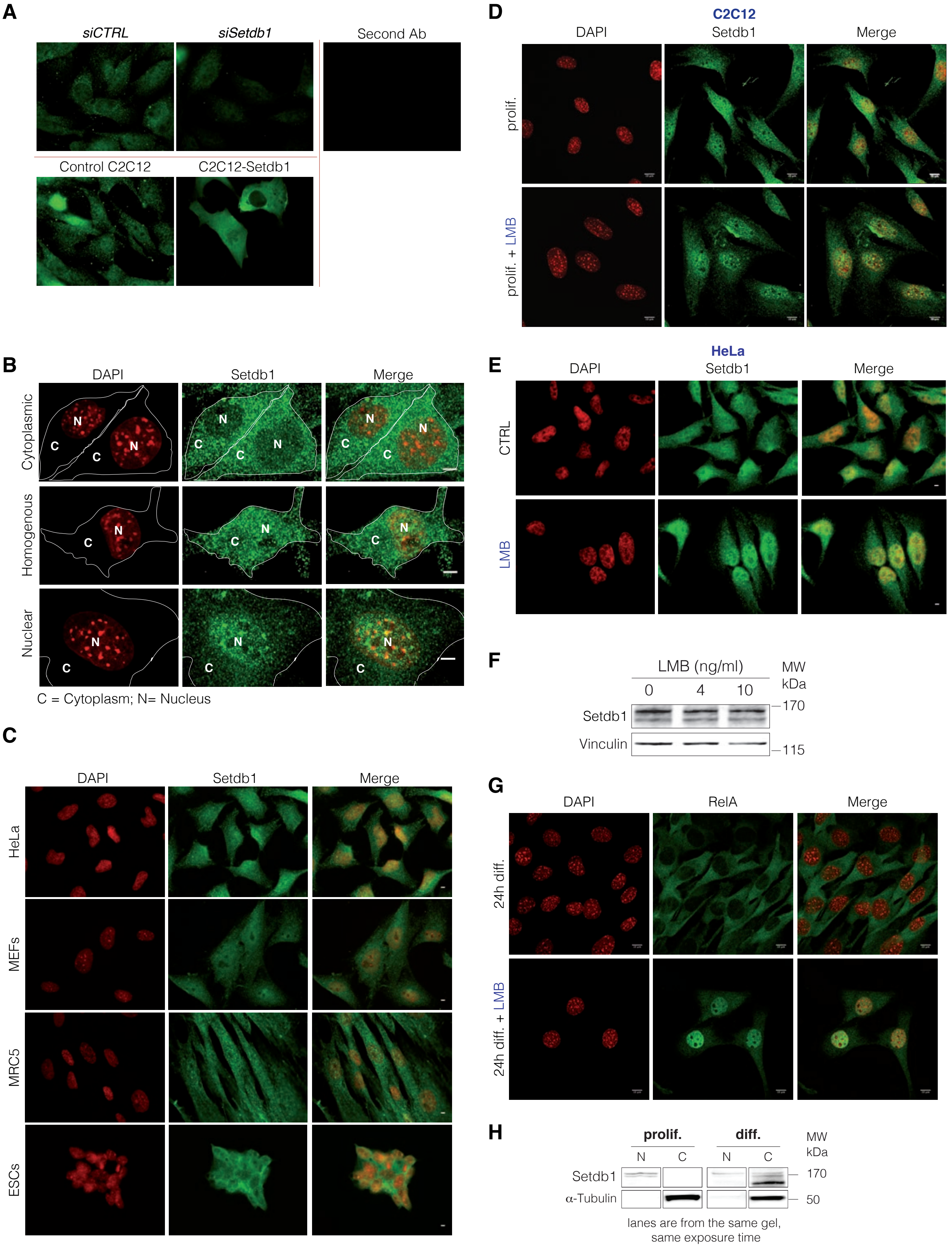

Figure S4: Nuclear and cytoplasmic localisation of Setdb1.

(A) Sepcificity if the Setdb1 immunofluorescence (IF) signal. Cellular Setdb1 (green) was detected by indirect IF in C2C12 myoblasts either transfected with a control siRNA (*siCTRL*) or Setdb1 siRNA (*siSetdb1*) (2 left upper panels); or in C2C12 cells stably overexpressing Setdb1 (C2C12-Setdb1) or an empty expression vector (control C2C12) (lower panels). The signal obtained with the secondary antibody is shown (Second Ab, upper right).

(**B)** Demonstration of different subcellular Setdb1 localisations. Enlargement of C2C12 myoblasts after indirect IF of Setdb1 (green). Setdb1 was classified as cytoplasmic, homogenous or nuclear if cells had the represented phenotypes. Nucleus and cytoplasm are marked as N and C respectively. DNA was stained with DAPI (red). Analysis was done by confocal microscopy. Scale bar = 5 μm.

(**C**) Setdb1 localisation in HeLa, MEFs, MRC5 and proliferating ESCs. Setdb1 (green) was stained by indirect IF. DNA was stained with DAPI (red). Scale bar =10 μm.

(**D**) Leptomycin B (LMB) changes Setdb1 localisation in proliferating C2C12 myoblasts. Indirect IF of Setdb1 (green) in cells, non-treated (prolif.) or treated with LMB for 18 h (prolif. + LMB). DNA was stained with DAPI (red). Analysis was done by confocal microscopy. Scale bar = 10 μm.

(**E**) Setdb1 localisation is changed after LMB treatment in HeLa cells. Indirect IF of Setdb1 (green) in HeLa cells, non-treated (CTRL) or treated with LMB for 10 h (LMB). DNA was stained with DAPI (red). Scale bar = 10 μm.

(**F**) Setdb1 global protein levels are not affected by LMB. WB analysis of Setdb1 in whole cell extracts from C2C12 myoblasts after 24 h of differentiation. Cells were in parallel treated with the indicated concentrations of LMB for the last 18 h. Vinculin; loading control.

(**G**) Nuclear export of RelA is diminished by LMB. Indirect IF of RelA (green) in C2C12 myoblasts after 24 h of differentiation (24 h diff.) and in parallel treated with LMB for the last 18 h (24 h diff. + LMB). DNA was co-stained with Dapi (red). Analysis was done by confocal microscopy. Scale bar 10 μm.

(**H**) Analysis of Setdb1 in nuclear (N) and cytoplasmic (C) fractions by WB in the nuclear (N) *versus* cytoplasmic (C) fractions of proliferating C2C12 myoblasts (prolif.) and after 24 h of differentiation (diff.). A typical experiment is shown. a-Tubulin, specific control for the cytoplasmic fraction. Note that the shown lanes were cut from the same gel.

All results are representative of a minimum of three independent experiments.

Figure S5, Beyer et al.

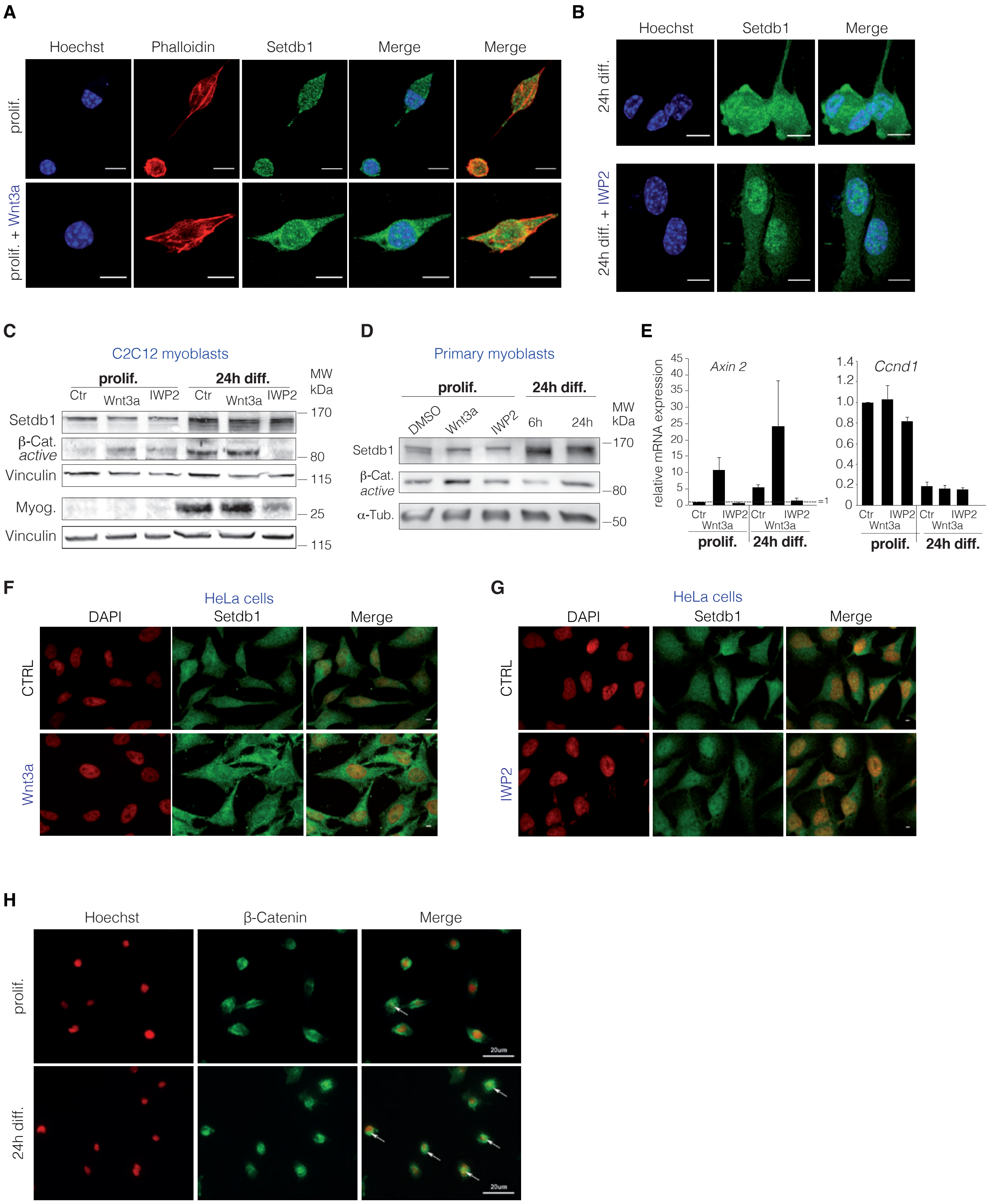

Figure S5: Setdb1 cellular localisation is dependent on Wnt3a signalling.

(**A**) Setdb1 localisation changes with increased Wnt3a signalling in proliferating primary mouse myoblasts. Cells were non-treated (prolif.) or stimulated with Wnt3a protein for 24 h (prolif. + Wnt3a). Indirect IF of Setdb1 (green) and confocal analysis was performed. Phalloidin (red) was used to co-stain for actin filaments and Hoechst to visualize DNA (blue). Scale bar = 10 μm.

(**B**) Inhibition of Wnt signalling restricts Setdb1 delocalisation in primary myoblasts. Cells were differentiated for 24 h and simultaneously treated with IWP2. IF was performed as described in (**A**).

(**C**) Setdb1, active b-Catenin and Myogenin protein levels were analysed in whole cell extracts of proliferating (prolif.) and 24 h differentiating (24 h diff.) C2C12 myoblasts. Cells were treated in parallel with Wnt3a or IWP2 for 24 h. Vinculin; loading control.

(**D**) Setdb1 and active b-Catenin protein levels were analysed in whole cell extracts from proliferating (prolif.) or 24 h differentiating (24h diff.) MuSC-derived primary mouse myoblasts. Cells were treated simultaneously with DMSO, Wnt3a or IWP2 for 24 h. α-Tubulin served as loading control.

(**E**) *Axin2* and *Ccnd1* relative mRNA expression analysis in C2C12 myoblasts. Cells were treated and cultured as described in C. Data are represented as fold change relative to proliferation values and normalised to *Cyclo A* and *TBP. Presented data are mean +/- SEM of a minimum of three independent experiments.*

(**F**) Setdb1 localisation changes with increased Wnt3a signalling in HeLa cells. Cells were non-treated (CTRL) or treated with Wnt3a for 24 h (Wnt3a). Indirect IF of Setdb1 (green) was performed. DNA was stained with DAPI (red). Scale bar = 10 μm.

(**G**) Setdb1 delocalisation is restricted when Wnt signalling is inhibited in HeLa cells. Indirect IF of Setdb1 (green) in cells, non-treated (CTRL) or treated with IWP2 for 24 h (IWP2). DNA was stained with DAPI (red). Scale bar = 10 μm.

(**H**) b-Catenin translocates to the nucleus during differentiation of primary myoblasts. Cells were proliferating (prolif.) or differentiating for 24 h. Indirect IF of b-Catenin (green) was performed. DNA was visualized by Hoechst. Scale bar = 20 μm.

*For **A** - **D**, **F** - **H**: Images are representatives of a minimum of three independent experiments.*

Figure S6, Beyer et al.

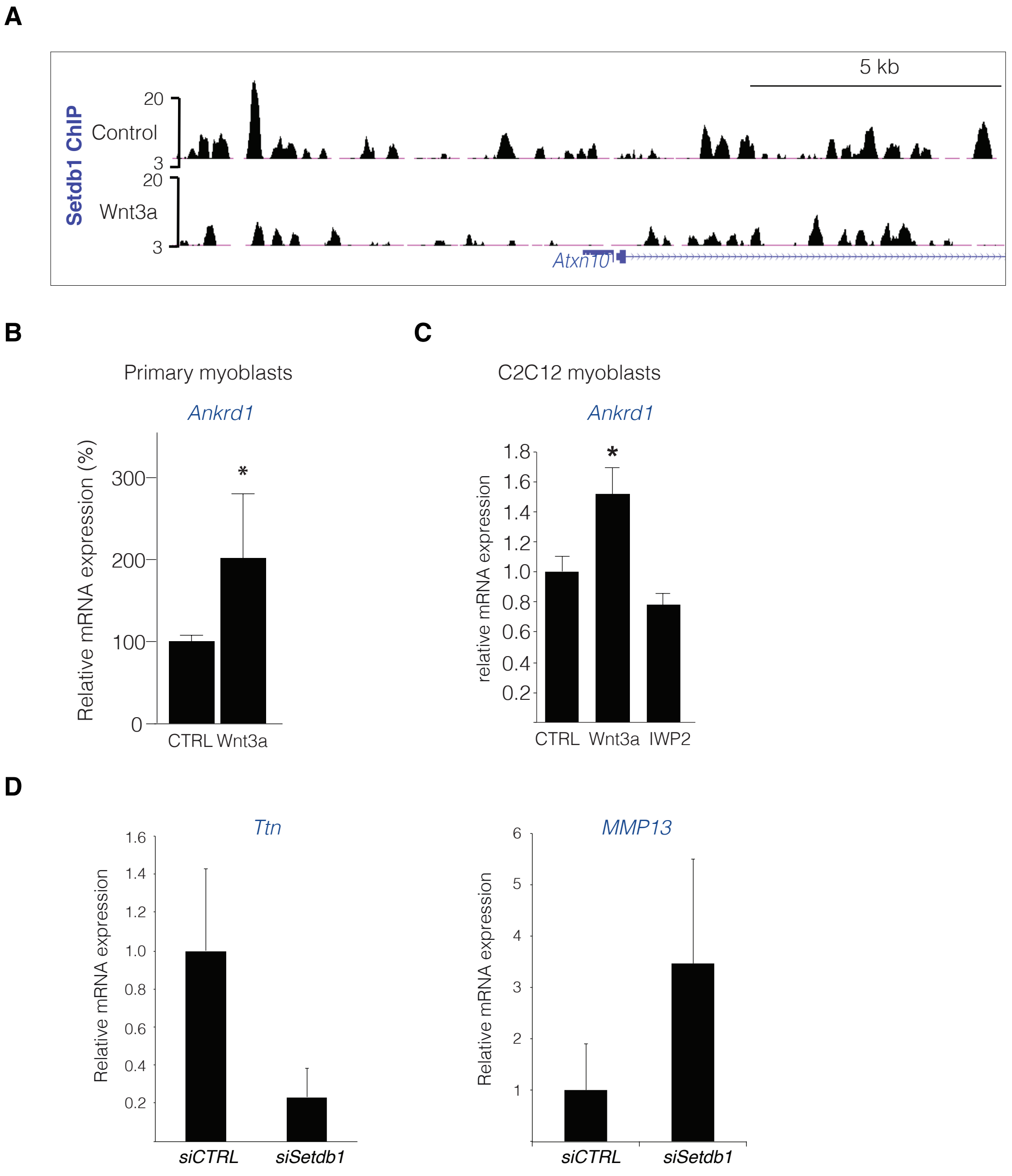

Figure S6: Wnt3a changes occupancy of Setdb1 at certain target genes.

(A) Genome Browser presentation of Setdb1 binding profile at Atxn10 promoter in proliferating C2C12 myoblasts non-treated (Control) or treated with Wnt3a for 24 h (Wnt3a).
(B) Ankrd1 mRNA increases in differentiating mouse primary myoblasts additionally treated with Wnt3a. Cells were differentiated for 24 h and simultaneously treated with Wnt3a.
(C) Ankrd1 mRNA changes in differentiating C2C12 myoblasts when Wnt signalling is additionally stimulated or inhibited. Cells were differentiated and simultaneously treated with Wnt3a or IWP2 for 24 h.
(D) Setdb1 knockdown in proliferating myoblasts decreases Ttn and increases MMP13 mRNA level. Proliferating C2C12 myoblasts were transfected with control siRNA (siCTRL) or Setdb1 siRNA (siSetdb1), as described for the RNA-seq assay. Relative mRNA expression levels of MMP13 and Ttn quantified by the normalized number of read counts for their transcripts in the RNA-seq data.

*For B and C: Data are represented as fold change relative to proliferation values and normalised to Cyclo A. Presented data are mean +/- SEM of a minimum of three independent experiments. *: p-values less than 0.05 and are considered significant.*
